# Pan-cancer analysis of whole genomes identifies driver rearrangements promoted by LINE-1 retrotransposition

**DOI:** 10.1038/s41588-019-0562-0

**Published:** 2020-02-05

**Authors:** Bernardo Rodriguez-Martin, Eva G. Alvarez, Adrian Baez-Ortega, Jorge Zamora, Fran Supek, Jonas Demeulemeester, Martin Santamarina, Young Seok Ju, Javier Temes, Daniel Garcia-Souto, Harald Detering, Yilong Li, Jorge Rodriguez-Castro, Ana Dueso-Barroso, Alicia L. Bruzos, Stefan C. Dentro, Miguel G. Blanco, Gianmarco Contino, Daniel Ardeljan, Marta Tojo, Nicola D. Roberts, Sonia Zumalave, Paul A. Edwards, Joachim Weischenfeldt, Montserrat Puiggròs, Zechen Chong, Ken Chen, Eunjung Alice Lee, Jeremiah A. Wala, Keiran M. Raine, Adam Butler, Sebastian M. Waszak, Fabio C. P. Navarro, Steven E. Schumacher, Jean Monlong, Francesco Maura, Niccolo Bolli, Guillaume Bourque, Mark Gerstein, Peter J. Park, David C. Wedge, Rameen Beroukhim, David Torrents, Jan O. Korbel, Iñigo Martincorena, Rebecca C. Fitzgerald, Peter Van Loo, Haig H. Kazazian, Kathleen H. Burns, PCAWG Structural Variation Working Group, Kadir C. Akdemir, Eva G. Alvarez, Adrian Baez-Ortega, Rameen Beroukhim, Paul C. Boutros, David D. L. Bowtell, Benedikt Brors, Kathleen H. Burns, Peter J. Campbell, Kin Chan, Ken Chen, Isidro Cortés-Ciriano, Ana Dueso-Barroso, Andrew J. Dunford, Paul A. Edwards, Xavier Estivill, Dariush Etemadmoghadam, Lars Feuerbach, J. Lynn Fink, Milana Frenkel-Morgenstern, Dale W. Garsed, Mark Gerstein, Dmitry A. Gordenin, David Haan, James E. Haber, Julian M. Hess, Barbara Hutter, Marcin Imielinski, David T. W. Jones, Young Seok Ju, Marat D. Kazanov, Leszek J. Klimczak, Youngil Koh, Jan O. Korbel, Kiran Kumar, Eunjung Alice Lee, Jake June-Koo Lee, Yilong Li, Andy G. Lynch, Geoff Macintyre, Florian Markowetz, Iñigo Martincorena, Alexander Martinez-Fundichely, Matthew Meyerson, Satoru Miyano, Hidewaki Nakagawa, Fabio C. P. Navarro, Stephan Ossowski, Peter J. Park, John V. Pearson, Montserrat Puiggròs, Karsten Rippe, Nicola D. Roberts, Steven A. Roberts, Bernardo Rodriguez-Martin, Steven E. Schumacher, Ralph Scully, Mark Shackleton, Nikos Sidiropoulos, Lina Sieverling, Chip Stewart, David Torrents, Jose M. C. Tubio, Izar Villasante, Nicola Waddell, Jeremiah A. Wala, Joachim Weischenfeldt, Lixing Yang, Xiaotong Yao, Sung-Soo Yoon, Jorge Zamora, Cheng-Zhong Zhang, Peter J. Campbell, Jose M. C. Tubio, PCAWG Consortium, Lauri A. Aaltonen, Federico Abascal, Adam Abeshouse, Hiroyuki Aburatani, David J. Adams, Nishant Agrawal, Keun Soo Ahn, Sung-Min Ahn, Hiroshi Aikata, Rehan Akbani, Kadir C. Akdemir, Hikmat Al-Ahmadie, Sultan T. Al-Sedairy, Fatima Al-Shahrour, Malik Alawi, Monique Albert, Kenneth Aldape, Ludmil B. Alexandrov, Adrian Ally, Kathryn Alsop, Eva G. Alvarez, Fernanda Amary, Samirkumar B. Amin, Brice Aminou, Ole Ammerpohl, Matthew J. Anderson, Yeng Ang, Davide Antonello, Pavana Anur, Samuel Aparicio, Elizabeth L. Appelbaum, Yasuhito Arai, Axel Aretz, Koji Arihiro, Shun-ichi Ariizumi, Joshua Armenia, Laurent Arnould, Sylvia Asa, Yassen Assenov, Gurnit Atwal, Sietse Aukema, J. Todd Auman, Miriam R. R. Aure, Philip Awadalla, Marta Aymerich, Gary D. Bader, Adrian Baez-Ortega, Matthew H. Bailey, Peter J. Bailey, Miruna Balasundaram, Saianand Balu, Pratiti Bandopadhayay, Rosamonde E. Banks, Stefano Barbi, Andrew P. Barbour, Jonathan Barenboim, Jill Barnholtz-Sloan, Hugh Barr, Elisabet Barrera, John Bartlett, Javier Bartolome, Claudio Bassi, Oliver F. Bathe, Daniel Baumhoer, Prashant Bavi, Stephen B. Baylin, Wojciech Bazant, Duncan Beardsmore, Timothy A. Beck, Sam Behjati, Andreas Behren, Beifang Niu, Cindy Bell, Sergi Beltran, Christopher Benz, Andrew Berchuck, Anke K. Bergmann, Erik N. Bergstrom, Benjamin P. Berman, Daniel M. Berney, Stephan H. Bernhart, Rameen Beroukhim, Mario Berrios, Samantha Bersani, Johanna Bertl, Miguel Betancourt, Vinayak Bhandari, Shriram G. Bhosle, Andrew V. Biankin, Matthias Bieg, Darell Bigner, Hans Binder, Ewan Birney, Michael Birrer, Nidhan K. Biswas, Bodil Bjerkehagen, Tom Bodenheimer, Lori Boice, Giada Bonizzato, Johann S. De Bono, Arnoud Boot, Moiz S. Bootwalla, Ake Borg, Arndt Borkhardt, Keith A. Boroevich, Ivan Borozan, Christoph Borst, Marcus Bosenberg, Mattia Bosio, Jacqueline Boultwood, Guillaume Bourque, Paul C. Boutros, G. Steven Bova, David T. Bowen, Reanne Bowlby, David D. L. Bowtell, Sandrine Boyault, Rich Boyce, Jeffrey Boyd, Alvis Brazma, Paul Brennan, Daniel S. Brewer, Arie B. Brinkman, Robert G. Bristow, Russell R. Broaddus, Jane E. Brock, Malcolm Brock, Annegien Broeks, Angela N. Brooks, Denise Brooks, Benedikt Brors, Søren Brunak, Timothy J. C. Bruxner, Alicia L. Bruzos, Alex Buchanan, Ivo Buchhalter, Christiane Buchholz, Susan Bullman, Hazel Burke, Birgit Burkhardt, Kathleen H. Burns, John Busanovich, Carlos D. Bustamante, Adam P. Butler, Atul J. Butte, Niall J. Byrne, Anne-Lise Børresen-Dale, Samantha J. Caesar-Johnson, Andy Cafferkey, Declan Cahill, Claudia Calabrese, Carlos Caldas, Fabien Calvo, Niedzica Camacho, Peter J. Campbell, Elias Campo, Cinzia Cantù, Shaolong Cao, Thomas E. Carey, Joana Carlevaro-Fita, Rebecca Carlsen, Ivana Cataldo, Mario Cazzola, Jonathan Cebon, Robert Cerfolio, Dianne E. Chadwick, Dimple Chakravarty, Don Chalmers, Calvin Wing Yiu Chan, Kin Chan, Michelle Chan-Seng-Yue, Vishal S. Chandan, David K. Chang, Stephen J. Chanock, Lorraine A. Chantrill, Aurélien Chateigner, Nilanjan Chatterjee, Kazuaki Chayama, Hsiao-Wei Chen, Jieming Chen, Ken Chen, Yiwen Chen, Zhaohong Chen, Andrew D. Cherniack, Jeremy Chien, Yoke-Eng Chiew, Suet-Feung Chin, Juok Cho, Sunghoon Cho, Jung Kyoon Choi, Wan Choi, Christine Chomienne, Zechen Chong, Su Pin Choo, Angela Chou, Angelika N. Christ, Elizabeth L. Christie, Eric Chuah, Carrie Cibulskis, Kristian Cibulskis, Sara Cingarlini, Peter Clapham, Alexander Claviez, Sean Cleary, Nicole Cloonan, Marek Cmero, Colin C. Collins, Ashton A. Connor, Susanna L. Cooke, Colin S. Cooper, Leslie Cope, Vincenzo Corbo, Matthew G. Cordes, Stephen M. Cordner, Isidro Cortés-Ciriano, Kyle Covington, Prue A. Cowin, Brian Craft, David Craft, Chad J. Creighton, Yupeng Cun, Erin Curley, Ioana Cutcutache, Karolina Czajka, Bogdan Czerniak, Rebecca A. Dagg, Ludmila Danilova, Maria Vittoria Davi, Natalie R. Davidson, Helen Davies, Ian J. Davis, Brandi N. Davis-Dusenbery, Kevin J. Dawson, Francisco M. De La Vega, Ricardo De Paoli-Iseppi, Timothy Defreitas, Angelo P. Dei Tos, Olivier Delaneau, John A. Demchok, Jonas Demeulemeester, German M. Demidov, Deniz Demircioğlu, Nening M. Dennis, Robert E. Denroche, Stefan C. Dentro, Nikita Desai, Vikram Deshpande, Amit G. Deshwar, Christine Desmedt, Jordi Deu-Pons, Noreen Dhalla, Neesha C. Dhani, Priyanka Dhingra, Rajiv Dhir, Anthony DiBiase, Klev Diamanti, Li Ding, Shuai Ding, Huy Q. Dinh, Luc Dirix, HarshaVardhan Doddapaneni, Nilgun Donmez, Michelle T. Dow, Ronny Drapkin, Oliver Drechsel, Ruben M. Drews, Serge Serge, Tim Dudderidge, Ana Dueso-Barroso, Andrew J. Dunford, Michael Dunn, Lewis Jonathan Dursi, Fraser R. Duthie, Ken Dutton-Regester, Jenna Eagles, Douglas F. Easton, Stuart Edmonds, Paul A. Edwards, Sandra E. Edwards, Rosalind A. Eeles, Anna Ehinger, Juergen Eils, Roland Eils, Adel El-Naggar, Matthew Eldridge, Kyle Ellrott, Serap Erkek, Georgia Escaramis, Shadrielle M. G. Espiritu, Xavier Estivill, Dariush Etemadmoghadam, Jorunn E. Eyfjord, Bishoy M. Faltas, Daiming Fan, Yu Fan, William C. Faquin, Claudiu Farcas, Matteo Fassan, Aquila Fatima, Francesco Favero, Nodirjon Fayzullaev, Ina Felau, Sian Fereday, Martin L. Ferguson, Vincent Ferretti, Lars Feuerbach, Matthew A. Field, J. Lynn Fink, Gaetano Finocchiaro, Cyril Fisher, Matthew W. Fittall, Anna Fitzgerald, Rebecca C. Fitzgerald, Adrienne M. Flanagan, Neil E. Fleshner, Paul Flicek, John A. Foekens, Kwun M. Fong, Nuno A. Fonseca, Christopher S. Foster, Natalie S. Fox, Michael Fraser, Scott Frazer, Milana Frenkel-Morgenstern, William Friedman, Joan Frigola, Catrina C. Fronick, Akihiro Fujimoto, Masashi Fujita, Masashi Fukayama, Lucinda A. Fulton, Robert S. Fulton, Mayuko Furuta, P. Andrew Futreal, Anja Füllgrabe, Stacey B. Gabriel, Steven Gallinger, Carlo Gambacorti-Passerini, Jianjiong Gao, Shengjie Gao, Levi Garraway, Øystein Garred, Erik Garrison, Dale W. Garsed, Nils Gehlenborg, Josep L. L. Gelpi, Joshy George, Daniela S. Gerhard, Clarissa Gerhauser, Jeffrey E. Gershenwald, Mark Gerstein, Moritz Gerstung, Gad Getz, Mohammed Ghori, Ronald Ghossein, Nasra H. Giama, Richard A. Gibbs, Bob Gibson, Anthony J. Gill, Pelvender Gill, Dilip D. Giri, Dominik Glodzik, Vincent J. Gnanapragasam, Maria Elisabeth Goebler, Mary J. Goldman, Carmen Gomez, Santiago Gonzalez, Abel Gonzalez-Perez, Dmitry A. Gordenin, James Gossage, Kunihito Gotoh, Ramaswamy Govindan, Dorthe Grabau, Janet S. Graham, Robert C. Grant, Anthony R. Green, Eric Green, Liliana Greger, Nicola Grehan, Sonia Grimaldi, Sean M. Grimmond, Robert L. Grossman, Adam Grundhoff, Gunes Gundem, Qianyun Guo, Manaswi Gupta, Shailja Gupta, Ivo G. Gut, Marta Gut, Jonathan Göke, Gavin Ha, Andrea Haake, David Haan, Siegfried Haas, Kerstin Haase, James E. Haber, Nina Habermann, Faraz Hach, Syed Haider, Natsuko Hama, Freddie C. Hamdy, Anne Hamilton, Mark P. Hamilton, Leng Han, George B. Hanna, Martin Hansmann, Nicholas J. Haradhvala, Olivier Harismendy, Ivon Harliwong, Arif O. Harmanci, Eoghan Harrington, Takanori Hasegawa, David Haussler, Steve Hawkins, Shinya Hayami, Shuto Hayashi, D. Neil Hayes, Stephen J. Hayes, Nicholas K. Hayward, Steven Hazell, Yao He, Allison P. Heath, Simon C. Heath, David Hedley, Apurva M. Hegde, David I. Heiman, Michael C. Heinold, Zachary Heins, Lawrence E. Heisler, Eva Hellstrom-Lindberg, Mohamed Helmy, Seong Gu Heo, Austin J. Hepperla, José María Heredia-Genestar, Carl Herrmann, Peter Hersey, Julian M. Hess, Holmfridur Hilmarsdottir, Jonathan Hinton, Satoshi Hirano, Nobuyoshi Hiraoka, Katherine A. Hoadley, Asger Hobolth, Ermin Hodzic, Jessica I. Hoell, Steve Hoffmann, Oliver Hofmann, Andrea Holbrook, Aliaksei Z. Holik, Michael A. Hollingsworth, Oliver Holmes, Robert A. Holt, Chen Hong, Eun Pyo Hong, Jongwhi H. Hong, Gerrit K. Hooijer, Henrik Hornshøj, Fumie Hosoda, Yong Hou, Volker Hovestadt, William Howat, Alan P. Hoyle, Ralph H. Hruban, Jianhong Hu, Taobo Hu, Xing Hua, Kuan-lin Huang, Mei Huang, Mi Ni Huang, Vincent Huang, Yi Huang, Wolfgang Huber, Thomas J. Hudson, Michael Hummel, Jillian A. Hung, David Huntsman, Ted R. Hupp, Jason Huse, Matthew R. Huska, Barbara Hutter, Carolyn M. Hutter, Daniel Hübschmann, Christine A. Iacobuzio-Donahue, Charles David Imbusch, Marcin Imielinski, Seiya Imoto, William B. Isaacs, Keren Isaev, Shumpei Ishikawa, Murat Iskar, S. M. Ashiqul Islam, Michael Ittmann, Sinisa Ivkovic, Jose M. G. Izarzugaza, Jocelyne Jacquemier, Valerie Jakrot, Nigel B. Jamieson, Gun Ho Jang, Se Jin Jang, Joy C. Jayaseelan, Reyka Jayasinghe, Stuart R. Jefferys, Karine Jegalian, Jennifer L. Jennings, Seung-Hyup Jeon, Lara Jerman, Yuan Ji, Wei Jiao, Peter A. Johansson, Amber L. Johns, Jeremy Johns, Rory Johnson, Todd A. Johnson, Clemency Jolly, Yann Joly, Jon G. Jonasson, Corbin D. Jones, David R. Jones, David T. W. Jones, Nic Jones, Steven J. M. Jones, Jos Jonkers, Young Seok Ju, Hartmut Juhl, Jongsun Jung, Malene Juul, Randi Istrup Juul, Sissel Juul, Natalie Jäger, Rolf Kabbe, Andre Kahles, Abdullah Kahraman, Vera B. Kaiser, Hojabr Kakavand, Sangeetha Kalimuthu, Christof von Kalle, Koo Jeong Kang, Katalin Karaszi, Beth Karlan, Rosa Karlić, Dennis Karsch, Katayoon Kasaian, Karin S. Kassahn, Hitoshi Katai, Mamoru Kato, Hiroto Katoh, Yoshiiku Kawakami, Jonathan D. Kay, Stephen H. Kazakoff, Marat D. Kazanov, Maria Keays, Electron Kebebew, Richard F. Kefford, Manolis Kellis, James G. Kench, Catherine J. Kennedy, Jules N. A. Kerssemakers, David Khoo, Vincent Khoo, Narong Khuntikeo, Ekta Khurana, Helena Kilpinen, Hark Kyun Kim, Hyung-Lae Kim, Hyung-Yong Kim, Hyunghwan Kim, Jaegil Kim, Jihoon Kim, Jong K. Kim, Youngwook Kim, Tari A. King, Wolfram Klapper, Kortine Kleinheinz, Leszek J. Klimczak, Stian Knappskog, Michael Kneba, Bartha M. Knoppers, Youngil Koh, Jan Komorowski, Daisuke Komura, Mitsuhiro Komura, Gu Kong, Marcel Kool, Jan O. Korbel, Viktoriya Korchina, Andrey Korshunov, Michael Koscher, Roelof Koster, Zsofia Kote-Jarai, Antonios Koures, Milena Kovacevic, Barbara Kremeyer, Helene Kretzmer, Markus Kreuz, Savitri Krishnamurthy, Dieter Kube, Kiran Kumar, Pardeep Kumar, Sushant Kumar, Yogesh Kumar, Ritika Kundra, Kirsten Kübler, Ralf Küppers, Jesper Lagergren, Phillip H. Lai, Peter W. Laird, Sunil R. Lakhani, Christopher M. Lalansingh, Emilie Lalonde, Fabien C. Lamaze, Adam Lambert, Eric Lander, Pablo Landgraf, Luca Landoni, Anita Langerød, Andrés Lanzós, Denis Larsimont, Erik Larsson, Mark Lathrop, Loretta M. S. Lau, Chris Lawerenz, Rita T. Lawlor, Michael S. Lawrence, Alexander J. Lazar, Ana Mijalkovic Lazic, Xuan Le, Darlene Lee, Donghoon Lee, Eunjung Alice Lee, Hee Jin Lee, Jake June-Koo Lee, Jeong-Yeon Lee, Juhee Lee, Ming Ta Michael Lee, Henry Lee-Six, Kjong-Van Lehmann, Hans Lehrach, Dido Lenze, Conrad R. Leonard, Daniel A. Leongamornlert, Ignaty Leshchiner, Louis Letourneau, Ivica Letunic, Douglas A. Levine, Lora Lewis, Tim Ley, Chang Li, Constance H. Li, Haiyan Irene Li, Jun Li, Lin Li, Shantao Li, Siliang Li, Xiaobo Li, Xiaotong Li, Xinyue Li, Yilong Li, Han Liang, Sheng-Ben Liang, Peter Lichter, Pei Lin, Ziao Lin, W. M. Linehan, Ole Christian Lingjærde, Dongbing Liu, Eric Minwei Liu, Fei-Fei Fei Liu, Fenglin Liu, Jia Liu, Xingmin Liu, Julie Livingstone, Dimitri Livitz, Naomi Livni, Lucas Lochovsky, Markus Loeffler, Georgina V. Long, Armando Lopez-Guillermo, Shaoke Lou, David N. Louis, Laurence B. Lovat, Yiling Lu, Yong-Jie Lu, Youyong Lu, Claudio Luchini, Ilinca Lungu, Xuemei Luo, Hayley J. Luxton, Andy G. Lynch, Lisa Lype, Cristina López, Carlos López-Otín, Eric Z. Ma, Yussanne Ma, Gaetan MacGrogan, Shona MacRae, Geoff Macintyre, Tobias Madsen, Kazuhiro Maejima, Andrea Mafficini, Dennis T. Maglinte, Arindam Maitra, Partha P. Majumder, Luca Malcovati, Salem Malikic, Giuseppe Malleo, Graham J. Mann, Luisa Mantovani-Löffler, Kathleen Marchal, Giovanni Marchegiani, Elaine R. Mardis, Adam A. Margolin, Maximillian G. Marin, Florian Markowetz, Julia Markowski, Jeffrey Marks, Tomas Marques-Bonet, Marco A. Marra, Luke Marsden, John W. M. Martens, Sancha Martin, Jose I. Martin-Subero, Iñigo Martincorena, Alexander Martinez-Fundichely, Yosef E. Maruvka, R. Jay Mashl, Charlie E. Massie, Thomas J. Matthew, Lucy Matthews, Erik Mayer, Simon Mayes, Michael Mayo, Faridah Mbabaali, Karen McCune, Ultan McDermott, Patrick D. McGillivray, Michael D. McLellan, John D. McPherson, John R. McPherson, Treasa A. McPherson, Samuel R. Meier, Alice Meng, Shaowu Meng, Andrew Menzies, Neil D. Merrett, Sue Merson, Matthew Meyerson, William Meyerson, Piotr A. Mieczkowski, George L. Mihaiescu, Sanja Mijalkovic, Tom Mikkelsen, Michele Milella, Linda Mileshkin, Christopher A. Miller, David K. Miller, Jessica K. Miller, Gordon B. Mills, Ana Milovanovic, Sarah Minner, Marco Miotto, Gisela Mir Arnau, Lisa Mirabello, Chris Mitchell, Thomas J. Mitchell, Satoru Miyano, Naoki Miyoshi, Shinichi Mizuno, Fruzsina Molnár-Gábor, Malcolm J. Moore, Richard A. Moore, Sandro Morganella, Quaid D. Morris, Carl Morrison, Lisle E. Mose, Catherine D. Moser, Ferran Muiños, Loris Mularoni, Andrew J. Mungall, Karen Mungall, Elizabeth A. Musgrove, Ville Mustonen, David Mutch, Francesc Muyas, Donna M. Muzny, Alfonso Muñoz, Jerome Myers, Ola Myklebost, Peter Möller, Genta Nagae, Adnan M. Nagrial, Hardeep K. Nahal-Bose, Hitoshi Nakagama, Hidewaki Nakagawa, Hiromi Nakamura, Toru Nakamura, Kaoru Nakano, Tannistha Nandi, Jyoti Nangalia, Mia Nastic, Arcadi Navarro, Fabio C. P. Navarro, David E. Neal, Gerd Nettekoven, Felicity Newell, Steven J. Newhouse, Yulia Newton, Alvin Wei Tian Ng, Anthony Ng, Jonathan Nicholson, David Nicol, Yongzhan Nie, G. Petur Nielsen, Morten Muhlig Nielsen, Serena Nik-Zainal, Michael S. Noble, Katia Nones, Paul A. Northcott, Faiyaz Notta, Brian D. O’Connor, Peter O’Donnell, Maria O’Donovan, Sarah O’Meara, Brian Patrick O’Neill, J. Robert O’Neill, David Ocana, Angelica Ochoa, Layla Oesper, Christopher Ogden, Hideki Ohdan, Kazuhiro Ohi, Lucila Ohno-Machado, Karin A. Oien, Akinyemi I. Ojesina, Hidenori Ojima, Takuji Okusaka, Larsson Omberg, Choon Kiat Ong, Stephan Ossowski, German Ott, B. F. Francis Ouellette, Christine P’ng, Marta Paczkowska, Salvatore Paiella, Chawalit Pairojkul, Marina Pajic, Qiang Pan-Hammarström, Elli Papaemmanuil, Irene Papatheodorou, Nagarajan Paramasivam, Ji Wan Park, Joong-Won Park, Keunchil Park, Kiejung Park, Peter J. Park, Joel S. Parker, Simon L. Parsons, Harvey Pass, Danielle Pasternack, Alessandro Pastore, Ann-Marie Patch, Iris Pauporté, Antonio Pea, John V. Pearson, Chandra Sekhar Pedamallu, Jakob Skou Pedersen, Paolo Pederzoli, Martin Peifer, Nathan A. Pennell, Charles M. Perou, Marc D. Perry, Gloria M. Petersen, Myron Peto, Nicholas Petrelli, Robert Petryszak, Stefan M. Pfister, Mark Phillips, Oriol Pich, Hilda A. Pickett, Todd D. Pihl, Nischalan Pillay, Sarah Pinder, Mark Pinese, Andreia V. Pinho, Esa Pitkänen, Xavier Pivot, Elena Piñeiro-Yáñez, Laura Planko, Christoph Plass, Paz Polak, Tirso Pons, Irinel Popescu, Olga Potapova, Aparna Prasad, Shaun R. Preston, Manuel Prinz, Antonia L. Pritchard, Stephenie D. Prokopec, Elena Provenzano, Xose S. Puente, Sonia Puig, Montserrat Puiggròs, Sergio Pulido-Tamayo, Gulietta M. Pupo, Colin A. Purdie, Michael C. Quinn, Raquel Rabionet, Janet S. Rader, Bernhard Radlwimmer, Petar Radovic, Benjamin Raeder, Keiran M. Raine, Manasa Ramakrishna, Kamna Ramakrishnan, Suresh Ramalingam, Benjamin J. Raphael, W. Kimryn Rathmell, Tobias Rausch, Guido Reifenberger, Jüri Reimand, Jorge Reis-Filho, Victor Reuter, Iker Reyes-Salazar, Matthew A. Reyna, Sheila M. Reynolds, Esther Rheinbay, Yasser Riazalhosseini, Andrea L. Richardson, Julia Richter, Matthew Ringel, Markus Ringnér, Yasushi Rino, Karsten Rippe, Jeffrey Roach, Lewis R. Roberts, Nicola D. Roberts, Steven A. Roberts, A. Gordon Robertson, Alan J. Robertson, Javier Bartolomé Rodriguez, Bernardo Rodriguez-Martin, F. Germán Rodríguez-González, Michael H. A. Roehrl, Marius Rohde, Hirofumi Rokutan, Gilles Romieu, Ilse Rooman, Tom Roques, Daniel Rosebrock, Mara Rosenberg, Philip C. Rosenstiel, Andreas Rosenwald, Edward W. Rowe, Romina Royo, Steven G. Rozen, Yulia Rubanova, Mark A. Rubin, Carlota Rubio-Perez, Vasilisa A. Rudneva, Borislav C. Rusev, Andrea Ruzzenente, Gunnar Rätsch, Radhakrishnan Sabarinathan, Veronica Y. Sabelnykova, Sara Sadeghi, S. Cenk Sahinalp, Natalie Saini, Mihoko Saito-Adachi, Gordon Saksena, Adriana Salcedo, Roberto Salgado, Leonidas Salichos, Richard Sallari, Charles Saller, Roberto Salvia, Michelle Sam, Jaswinder S. Samra, Francisco Sanchez-Vega, Chris Sander, Grant Sanders, Rajiv Sarin, Iman Sarrafi, Aya Sasaki-Oku, Torill Sauer, Guido Sauter, Robyn P. M. Saw, Maria Scardoni, Christopher J. Scarlett, Aldo Scarpa, Ghislaine Scelo, Dirk Schadendorf, Jacqueline E. Schein, Markus B. Schilhabel, Matthias Schlesner, Thorsten Schlomm, Heather K. Schmidt, Sarah-Jane Schramm, Stefan Schreiber, Nikolaus Schultz, Steven E. Schumacher, Roland F. Schwarz, Richard A. Scolyer, David Scott, Ralph Scully, Raja Seethala, Ayellet V. Segre, Iris Selander, Colin A. Semple, Yasin Senbabaoglu, Subhajit Sengupta, Elisabetta Sereni, Stefano Serra, Dennis C. Sgroi, Mark Shackleton, Nimish C. Shah, Sagedeh Shahabi, Catherine A. Shang, Ping Shang, Ofer Shapira, Troy Shelton, Ciyue Shen, Hui Shen, Rebecca Shepherd, Ruian Shi, Yan Shi, Yu-Jia Shiah, Tatsuhiro Shibata, Juliann Shih, Eigo Shimizu, Kiyo Shimizu, Seung Jun Shin, Yuichi Shiraishi, Tal Shmaya, Ilya Shmulevich, Solomon I. Shorser, Charles Short, Raunak Shrestha, Suyash S. Shringarpure, Craig Shriver, Shimin Shuai, Nikos Sidiropoulos, Reiner Siebert, Anieta M. Sieuwerts, Lina Sieverling, Sabina Signoretti, Katarzyna O. Sikora, Michele Simbolo, Ronald Simon, Janae V. Simons, Jared T. Simpson, Peter T. Simpson, Samuel Singer, Nasa Sinnott-Armstrong, Payal Sipahimalani, Tara J. Skelly, Marcel Smid, Jaclyn Smith, Karen Smith-McCune, Nicholas D. Socci, Heidi J. Sofia, Matthew G. Soloway, Lei Song, Anil K. Sood, Sharmila Sothi, Christos Sotiriou, Cameron M. Soulette, Paul N. Span, Paul T. Spellman, Nicola Sperandio, Andrew J. Spillane, Oliver Spiro, Jonathan Spring, Johan Staaf, Peter F. Stadler, Peter Staib, Stefan G. Stark, Lucy Stebbings, Ólafur Andri Stefánsson, Oliver Stegle, Lincoln D. Stein, Alasdair Stenhouse, Chip Stewart, Stephan Stilgenbauer, Miranda D. Stobbe, Michael R. Stratton, Jonathan R. Stretch, Adam J. Struck, Joshua M. Stuart, Henk G. Stunnenberg, Hong Su, Xiaoping Su, Ren X. Sun, Stephanie Sungalee, Hana Susak, Akihiro Suzuki, Fred Sweep, Monika Szczepanowski, Holger Sültmann, Takashi Yugawa, Angela Tam, David Tamborero, Benita Kiat Tee Tan, Donghui Tan, Patrick Tan, Hiroko Tanaka, Hirokazu Taniguchi, Tomas J. Tanskanen, Maxime Tarabichi, Roy Tarnuzzer, Patrick Tarpey, Morgan L. Taschuk, Kenji Tatsuno, Simon Tavaré, Darrin F. Taylor, Amaro Taylor-Weiner, Jon W. Teague, Bin Tean Teh, Varsha Tembe, Javier Temes, Kevin Thai, Sarah P. Thayer, Nina Thiessen, Gilles Thomas, Sarah Thomas, Alan Thompson, Alastair M. Thompson, John F. F. Thompson, R. Houston Thompson, Heather Thorne, Leigh B. Thorne, Adrian Thorogood, Grace Tiao, Nebojsa Tijanic, Lee E. Timms, Roberto Tirabosco, Marta Tojo, Stefania Tommasi, Christopher W. Toon, Umut H. Toprak, David Torrents, Giampaolo Tortora, Jörg Tost, Yasushi Totoki, David Townend, Nadia Traficante, Isabelle Treilleux, Jean-Rémi Trotta, Lorenz H. P. Trümper, Ming Tsao, Tatsuhiko Tsunoda, Jose M. C. Tubio, Olga Tucker, Richard Turkington, Daniel J. Turner, Andrew Tutt, Masaki Ueno, Naoto T. Ueno, Christopher Umbricht, Husen M. Umer, Timothy J. Underwood, Lara Urban, Tomoko Urushidate, Tetsuo Ushiku, Liis Uusküla-Reimand, Alfonso Valencia, David J. Van Den Berg, Steven Van Laere, Peter Van Loo, Erwin G. Van Meir, Gert G. Van den Eynden, Theodorus Van der Kwast, Naveen Vasudev, Miguel Vazquez, Ravikiran Vedururu, Umadevi Veluvolu, Shankar Vembu, Lieven P. C. Verbeke, Peter Vermeulen, Clare Verrill, Alain Viari, David Vicente, Caterina Vicentini, K. VijayRaghavan, Juris Viksna, Ricardo E. Vilain, Izar Villasante, Anne Vincent-Salomon, Tapio Visakorpi, Douglas Voet, Paresh Vyas, Ignacio Vázquez-García, Nick M. Waddell, Nicola Waddell, Claes Wadelius, Lina Wadi, Rabea Wagener, Jeremiah A. Wala, Jian Wang, Jiayin Wang, Linghua Wang, Qi Wang, Wenyi Wang, Yumeng Wang, Zhining Wang, Paul M. Waring, Hans-Jörg Warnatz, Jonathan Warrell, Anne Y. Warren, Sebastian M. Waszak, David C. Wedge, Dieter Weichenhan, Paul Weinberger, John N. Weinstein, Joachim Weischenfeldt, Daniel J. Weisenberger, Ian Welch, Michael C. Wendl, Johannes Werner, Justin P. Whalley, David A. Wheeler, Hayley C. Whitaker, Dennis Wigle, Matthew D. Wilkerson, Ashley Williams, James S. Wilmott, Gavin W. Wilson, Julie M. Wilson, Richard K. Wilson, Boris Winterhoff, Jeffrey A. Wintersinger, Maciej Wiznerowicz, Stephan Wolf, Bernice H. Wong, Tina Wong, Winghing Wong, Youngchoon Woo, Scott Wood, Bradly G. Wouters, Adam J. Wright, Derek W. Wright, Mark H. Wright, Chin-Lee Wu, Dai-Ying Wu, Guanming Wu, Jianmin Wu, Kui Wu, Yang Wu, Zhenggang Wu, Liu Xi, Tian Xia, Qian Xiang, Xiao Xiao, Rui Xing, Heng Xiong, Qinying Xu, Yanxun Xu, Hong Xue, Shinichi Yachida, Sergei Yakneen, Rui Yamaguchi, Takafumi N. Yamaguchi, Masakazu Yamamoto, Shogo Yamamoto, Hiroki Yamaue, Fan Yang, Huanming Yang, Jean Y. Yang, Liming Yang, Lixing Yang, Shanlin Yang, Tsun-Po Yang, Yang Yang, Xiaotong Yao, Marie-Laure Yaspo, Lucy Yates, Christina Yau, Chen Ye, Kai Ye, Venkata D. Yellapantula, Christopher J. Yoon, Sung-Soo Yoon, Fouad Yousif, Jun Yu, Kaixian Yu, Willie Yu, Yingyan Yu, Ke Yuan, Yuan Yuan, Denis Yuen, Christina K. Yung, Olga Zaikova, Jorge Zamora, Marc Zapatka, Jean C. Zenklusen, Thorsten Zenz, Nikolajs Zeps, Cheng-Zhong Zhang, Fan Zhang, Hailei Zhang, Hongwei Zhang, Hongxin Zhang, Jiashan Zhang, Jing Zhang, Junjun Zhang, Xiuqing Zhang, Xuanping Zhang, Yan Zhang, Zemin Zhang, Zhongming Zhao, Liangtao Zheng, Xiuqing Zheng, Wanding Zhou, Yong Zhou, Bin Zhu, Hongtu Zhu, Jingchun Zhu, Shida Zhu, Lihua Zou, Xueqing Zou, Anna deFazio, Nicholas van As, Carolien H. M. van Deurzen, Marc J. van de Vijver, L. van’t Veer, Christian von Mering

**Affiliations:** 1 Department of Zoology, Genetics and Physical Anthropology, Universidade de Santiago de Compostela, Santiago de Compostela, Spain; 2 Biomedical Research Centre (CINBIO), University of Vigo, Vigo, Spain; 3 Centre for Research in Molecular Medicine and Chronic Diseases (CIMUS), Universidade de Santiago de Compostela, Santiago de Compostela, Spain; 4 Transmissible Cancer Group, Department of Veterinary Medicine, University of Cambridge, Cambridge, UK; 5 The Biomedical Research Centre (CINBIO), Universidade de Vigo, Vigo, Spain; 6 Wellcome Sanger Institute, Wellcome Genome Campus, Cambridge, UK; 7 Genome Data Science, Institute for Research in Biomedicine (IRB Barcelona), The Barcelona Institute of Science and Technology (BIST), Barcelona, Spain; 8 Institucio Catalana de Recerca i Estudis Avançats (ICREA), Barcelona, Spain; 9 The Francis Crick Institute, London, UK; 10 Department of Human Genetics, University of Leuven, Leuven, Belgium; 11 Genomes and Disease, Centre for Research in Molecular Medicine and Chronic Diseases (CIMUS), Universidade de Santiago de Compostela, Santiago de Compostela, Spain; 12 Graduate School of Medical Science and Engineering, Korea Advanced Institute of Science and Technology, Daejeon, South Korea; 13 Cancer Ageing and Somatic Mutation Programme, Wellcome Sanger Institute, Cambridge, UK; 14 Department of Biochemistry, Genetics and Immunology, University of Vigo, Vigo, Spain; 15 Galicia Sur Health Research Institute, Vigo, Spain; 16 Faculty of Science and Technology, University of Vic—Central University of Catalonia (UVic-UCC), Vic, Spain; 17 Barcelona Supercomputing Center (BSC), Barcelona, Spain; 18 Experimental Cancer Genetics, Wellcome Sanger Institute, Cambridge, UK; 19 Oxford Big Data Institute, University of Oxford, Oxford, UK; 20 DNA Repair and Genome Integrity, Centre for Research in Molecular Medicine and Chronic Diseases (CIMUS), Universidade de Santiago de Compostela, Santiago de Compostela, Spain; 21 Department of Biochemistry and Molecular Biology, Universidade de Santiago de Compostela, Santiago de Compostela, Spain; 22 Medical Research Council (MRC) Cancer Unit, University of Cambridge, Cambridge, UK; 23 Department of Genetic Medicine, Johns Hopkins University School of Medicine, Baltimore, Baltimore, MD USA; 24 University of Cambridge, Cambridge, UK; 25 Li Ka Shing Centre, Cancer Research UK Cambridge Institute, University of Cambridge, Cambridge, UK; 26 European Molecular Biology Laboratory (EMBL), Genome Biology Unit, Heidelberg, Germany; 27 Finsen Laboratory and Biotech Research & Innovation Centre (BRIC), University of Copenhagen, Copenhagen, Denmark; 28 Department of Urology, Charité Universitätsmedizin Berlin, Berlin, Germany; 29 Department of Bioinformatics and Computational Biology, The University of Texas MD Anderson Cancer Center, Houston, TX USA; 30 Department of Genetics and Informatics Institute, University of Alabama at Birmingham (UAB) School of Medicine, Birmingham, AL USA; 31 University of Texas MD Anderson Cancer Center, Houston, TX USA; 32 Division of Genetics and Genomics, Boston Children’s Hospital, Harvard Medical School, Boston, MA USA; 33 The Broad Institute of Harvard and MIT, Cambridge, MA USA; 34 Department of Cancer Biology, Dana-Farber Cancer Institute, Boston, MA USA; 35 Department of Medical Oncology, Dana-Farber Cancer Institute, Boston, MA USA; 36 Harvard Medical School, Boston, MA USA; 37 Program in Computational Biology and Bioinformatics, Yale University, New Haven, CT USA; 38 Department of Molecular Biophysics and Biochemistry, Yale University, New Haven, CT USA; 39 Department of Computer Science, Yale University, New Haven, CT USA; 40 Department of Human Genetics, McGill University, Montreal, Québec Canada; 41 Department of Oncology and Onco-Hematology, University of Milan, Milan, Italy; 42 Department of Medical Oncology and Hematology, Fondazione IRCCS Istituto Nazionale dei Tumori, Milan, Italy; 43 Canadian Center for Computational Genomics, McGill University, Montreal, Quebec, Canada; 44 Department of Biomedical Informatics, Harvard Medical School, Boston, MA USA; 45 Ludwig Center at Harvard, Boston, MA USA; 46 Oxford NIHR Biomedical Research Centre, Oxford, UK; 47 European Molecular Biology Laboratory, European Bioinformatics Institute (EMBL-EBI), Cambridge, UK; 48 Department of Pathology, Johns Hopkins University School of Medicine, Baltimore, Baltimore, MD USA; 49 McKusick-Nathans Institute of Genetic Medicine, Sidney Kimmel Comprehensive Cancer Center at Johns Hopkins University School of Medicine, Baltimore, MD USA; 51 Department of Haematology, University of Cambridge, Cambridge, UK; 53 Computational Biology Program, Ontario Institute for Cancer Research, Toronto, Ontario Canada; 54 Department of Medical Biophysics, University of Toronto, Toronto, Ontario Canada; 55 Department of Pharmacology, University of Toronto, Toronto, Ontario Canada; 56 University of California Los Angeles, Los Angeles, CA USA; 57 Peter MacCallum Cancer Centre, Melbourne, Victoria Australia; 58 Sir Peter MacCallum Department of Oncology, University of Melbourne, Melbourne, Victoria Australia; 61 Division of Applied Bioinformatics, German Cancer Research Center (DKFZ), Heidelberg, Germany; 59 National Center for Tumor Diseases (NCT) Heidelberg, Heidelberg, Germany; 60 Johns Hopkins School of Medicine, Baltimore, MD USA; 62 Department of Biochemistry, Microbiology and Immunology, Faculty of Medicine, University of Ottawa, Ottawa, Ontario Canada; 63 Centre for Molecular Science Informatics, Department of Chemistry, University of Cambridge, Cambridge, UK; 64 Ludwig Center, Harvard Medical School, Boston, MA USA; 65 Broad Institute of MIT and Harvard, Cambridge, MA USA; 66 Sidra Medicine, Doha, Qatar; 67 Sir Peter MacCallum Department of Oncology, The University of Melbourne, Melbourne, Victoria Australia; 68 Queensland Centre for Medical Genomics, Institute for Molecular Bioscience, The University of Queensland, St Lucia, Queensland Australia; 69 The Azrieli Faculty of Medicine, Bar-Ilan University, Safed, Israel; 70 Genome Integrity and Structural Biology Laboratory, National Institute of Environmental Health Sciences (NIEHS), Durham, NC USA; 71 Biomolecular Engineering Department, University of California, Santa Cruz, Santa Cruz, CA USA; 72 Brandeis University, Waltham, MA USA; 73 Massachusetts General Hospital Center for Cancer Research, Charlestown, MA USA; 74 German Cancer Consortium (DKTK), Heidelberg, Germany; 75 Heidelberg Center for Personalized Oncology (DKFZ-HIPO), German Cancer Research Center (DKFZ), Heidelberg, Germany; 76 New York Genome Center, New York, NY USA; 77 Weill Cornell Medicine, New York, NY USA; 78 Hopp Children’s Cancer Center (KiTZ), Heidelberg, Germany; 79 Pediatric Glioma Research Group, German Cancer Research Center (DKFZ), Heidelberg, Germany; 80 Korea Advanced Institute of Science and Technology, Daejeon, South Korea; 81 Skolkovo Institute of Science and Technology, Moscow, Russia; 82 A. A. Kharkevich Institute of Information Transmission Problems, Moscow, Russia; 83 Dmitry Rogachev National Research Center of Pediatric Hematology, Oncology and Immunology, Moscow, Russia; 84 Integrative Bioinformatics Support Group, National Institute of Environmental Health Sciences (NIEHS), Durham, NC USA; 85 Center For Medical Innovation, Seoul National University Hospital, Seoul, South Korea; 86 Department of Internal Medicine, Seoul National University Hospital, Seoul, South Korea; 87 Cancer Research UK Cambridge Institute, University of Cambridge, Cambridge, UK; 88 School of Medicine, University of St Andrews, St Andrews, UK; 89 School of Mathematics and Statistics, University of St Andrews, St Andrews, UK; 90 Department of Physiology and Biophysics, Weill Cornell Medicine, New York, NY USA; 91 Englander Institute for Precision Medicine, Weill Cornell Medicine, New York, NY USA; 92 Institute for Computational Biomedicine, Weill Cornell Medicine, New York, NY USA; 93 Dana-Farber Cancer Institute, Boston, MA USA; 94 Department of Medical Oncology, Inselspital, University Hospital and University of Bern, Bern, Switzerland; 95 Department of Pathology, The University of Melbourne, Melbourne, Victoria Australia; 96 The Institute of Medical Science, The University of Tokyo, Tokyo, Japan; 97 RIKEN Center for Integrative Medical Sciences, Yokohama, Japan; 98 Centre for Genomic Regulation (CRG), The Barcelona Institute of Science and Technology, Barcelona, Spain; 99 Institute of Medical Genetics and Applied Genomics, University of Tübingen, Tübingen, Germany; 100 Universitat Pompeu Fabra (UPF), Barcelona, Spain; 101 Department of Genetics and Computational Biology, QIMR Berghofer Medical Research Institute, Brisbane, Australia; 102 Institute for Molecular Bioscience, University of Queensland, St Lucia, Brisbane, Queensland Australia; 103 German Cancer Research Center (DKFZ), Heidelberg, Germany; 104 School of Molecular Biosciences and Center for Reproductive Biology, Washington State University, Pullman, WA USA; 105 Cancer Research Institute, Beth Israel Deaconess Medical Center, Boston, MA USA; 106 Peter MacCallum Cancer Centre and University of Melbourne, Melbourne, Victoria Australia; 107 Faculty of Biosciences, Heidelberg University, Heidelberg, Germany; 108 Ben May Department for Cancer Research, Department of Human Genetics, The University of Chicago, Chicago, IL USA; 109 Tri-institutional PhD Program of Computational Biology and Medicine, Weill Cornell Medicine, New York, NY USA; 200 Applied Tumor Genomics Research Program, Research Programs Unit, University of Helsinki, Helsinki, Finland; 201 Wellcome Sanger Institute, Wellcome Genome Campus, Hinxton, UK; 202 Memorial Sloan Kettering Cancer Center, New York, NY USA; 203 Genome Science Division, Research Center for Advanced Science and Technology, University of Tokyo, Tokyo, Japan; 204 Department of Surgery, University of Chicago, Chicago, IL USA; 205 Department of Surgery, Division of Hepatobiliary and Pancreatic Surgery, School of Medicine, Keimyung University Dongsan Medical Center, Daegu, South Korea; 206 Department of Oncology, Gil Medical Center, Gachon University, Incheon, South Korea; 207 Hiroshima University, Hiroshima, Japan; 208 Department of Bioinformatics and Computational Biology, The University of Texas MD Anderson Cancer Center, Houston, TX USA; 209 University of Texas MD Anderson Cancer Center, Houston, TX USA; 210 King Faisal Specialist Hospital and Research Centre, Al Maather, Riyadh, Saudi Arabia; 211 Bioinformatics Unit, Spanish National Cancer Research Centre (CNIO), Madrid, Spain; 212 Bioinformatics Core Facility, University Medical Center Hamburg, Hamburg, Germany; 213 Heinrich Pette Institute, Leibniz Institute for Experimental Virology, Hamburg, Germany; 214 Ontario Tumour Bank, Ontario Institute for Cancer Research, Toronto, ON Canada; 215 Department of Pathology, The University of Texas MD Anderson Cancer Center, Houston, TX USA; 216 Laboratory of Pathology, Center for Cancer Research, National Cancer Institute, Bethesda, MD USA; 217 Department of Cellular and Molecular Medicine and Department of Bioengineering, University of California San Diego, La Jolla, CA USA; 218 UC San Diego Moores Cancer Center, San Diego, CA USA; 219 Canada’s Michael Smith Genome Sciences Centre, BC Cancer, Vancouver, BC Canada; 220 Sir Peter MacCallum Department of Oncology, Peter MacCallum Cancer Centre, University of Melbourne, Melbourne, VIC Australia; 221 Centre for Research in Molecular Medicine and Chronic Diseases (CiMUS), Universidade de Santiago de Compostela, Santiago de Compostela, Spain; 222 Department of Zoology, Genetics and Physical Anthropology, (CiMUS), Universidade de Santiago de Compostela, Santiago de Compostela, Spain; 223 The Biomedical Research Centre (CINBIO), Universidade de Vigo, Vigo, Spain; 224 Royal National Orthopaedic Hospital - Bolsover, London, UK; 225 Department of Genomic Medicine, The University of Texas MD Anderson Cancer Center, Houston, TX USA; 226 Quantitative and Computational Biosciences Graduate Program, Baylor College of Medicine, Houston, TX USA; 227 The Jackson Laboratory for Genomic Medicine, Farmington, CT USA; 228 Genome Informatics Program, Ontario Institute for Cancer Research, Toronto, ON Canada; 229 Institute of Human Genetics, Christian-Albrechts-University, Kiel, Germany; 230 Institute of Human Genetics, Ulm University and Ulm University Medical Center, Ulm, Germany; 231 Queensland Centre for Medical Genomics, Institute for Molecular Bioscience, University of Queensland, St. Lucia, Brisbane, QLD Australia; 232 Salford Royal NHS Foundation Trust, Salford, UK; 233 Department of Surgery, Pancreas Institute, University and Hospital Trust of Verona, Verona, Italy; 234 Molecular and Medical Genetics, OHSU Knight Cancer Institute, Oregon Health and Science University, Portland, OR USA; 235 Department of Molecular Oncology, BC Cancer Research Centre, Vancouver, BC Canada; 236 The McDonnell Genome Institute at Washington University, St. Louis, MO USA; 237 University College London, London, UK; 238 Division of Cancer Genomics, National Cancer Center Research Institute, National Cancer Center, Tokyo, Japan; 239 DLR Project Management Agency, Bonn, Germany; 240 Tokyo Women’s Medical University, Tokyo, Japan; 241 Center for Molecular Oncology, Memorial Sloan Kettering Cancer Center, New York, NY USA; 242 Los Alamos National Laboratory, Los Alamos, NM USA; 243 Department of Pathology, University Health Network, Toronto General Hospital, Toronto, ON Canada; 244 Nottingham University Hospitals NHS Trust, Nottingham, UK; 245 Epigenomics and Cancer Risk Factors, German Cancer Research Center (DKFZ), Heidelberg, Germany; 246 Computational Biology Program, Ontario Institute for Cancer Research, Toronto, ON Canada; 247 Department of Molecular Genetics, University of Toronto, Toronto, ON Canada; 248 Vector Institute, Toronto, ON Canada; 249 Hematopathology Section, Institute of Pathology, Christian-Albrechts-University, Kiel, Germany; 250 Department of Pathology and Laboratory Medicine, School of Medicine, University of North Carolina at Chapel Hill, Chapel Hill, NC USA; 251 Department of Cancer Genetics, Institute for Cancer Research, Oslo University Hospital, The Norwegian Radium Hospital, Oslo, Norway; 252 Pathology, Hospital Clinic, Institut d’Investigacions Biomèdiques August Pi i Sunyer (IDIBAPS), University of Barcelona, Barcelona, Spain; 253 Department of Veterinary Medicine, Transmissible Cancer Group, University of Cambridge, Cambridge, UK; 254 Alvin J. Siteman Cancer Center, Washington University School of Medicine, St. Louis, MO USA; 255 Wolfson Wohl Cancer Research Centre, Institute of Cancer Sciences, University of Glasgow, Glasgow, UK; 256 Lineberger Comprehensive Cancer Center, University of North Carolina at Chapel Hill, Chapel Hill, NC USA; 257 Broad Institute of MIT and Harvard, Cambridge, MA USA; 258 Dana-Farber/Boston Children’s Cancer and Blood Disorders Center, Boston, MA USA; 259 Department of Pediatrics, Harvard Medical School, Boston, MA USA; 260 Leeds Institute of Medical Research @ St. James’s, University of Leeds, St. James’s University Hospital, Leeds, UK; 261 Department of Pathology and Diagnostics, University and Hospital Trust of Verona, Verona, Italy; 262 Department of Surgery, Princess Alexandra Hospital, Brisbane, QLD Australia; 263 Surgical Oncology Group, Diamantina Institute, University of Queensland, Brisbane, QLD Australia; 264 Department of Population and Quantitative Health Sciences, Case Western Reserve University School of Medicine, Cleveland, OH USA; 265 Research Health Analytics and Informatics, University Hospitals Cleveland Medical Center, Cleveland, OH USA; 266 Gloucester Royal Hospital, Gloucester, UK; 267 European Molecular Biology Laboratory, European Bioinformatics Institute (EMBL-EBI), Cambridge, UK; 268 Diagnostic Development, Ontario Institute for Cancer Research, Toronto, ON Canada; 269 Barcelona Supercomputing Center (BSC), Barcelona, Spain; 270 Arnie Charbonneau Cancer Institute, University of Calgary, Calgary, AB Canada; 271 Departments of Surgery and Oncology, University of Calgary, Calgary, AB Canada; 272 Department of Pathology, Oslo University Hospital, The Norwegian Radium Hospital, Oslo, Norway; 273 PanCuRx Translational Research Initiative, Ontario Institute for Cancer Research, Toronto, ON Canada; 274 Department of Oncology, Sidney Kimmel Comprehensive Cancer Center at Johns Hopkins University School of Medicine, Baltimore, MD USA; 275 University Hospital Southampton NHS Foundation Trust, Southampton, UK; 276 Royal Stoke University Hospital, Stoke-on-Trent, UK; 277 Genome Sequence Informatics, Ontario Institute for Cancer Research, Toronto, ON Canada; 278 Human Longevity Inc, San Diego, CA USA; 279 Olivia Newton-John Cancer Research Institute, La Trobe University, Heidelberg, VIC Australia; 280 Computer Network Information Center, Chinese Academy of Sciences, Beijing, China; 281 Genome Canada, Ottawa, ON Canada; 282 CNAG-CRG, Centre for Genomic Regulation (CRG), Barcelona Institute of Science and Technology (BIST), Barcelona, Spain; 283 Universitat Pompeu Fabra (UPF), Barcelona, Spain; 284 Buck Institute for Research on Aging, Novato, CA USA; 285 Duke University Medical Center, Durham, NC USA; 286 Department of Human Genetics, Hannover Medical School, Hannover, Germany; 287 Center for Bioinformatics and Functional Genomics, Cedars-Sinai Medical Center, Los Angeles, CA USA; 288 Department of Biomedical Sciences, Cedars-Sinai Medical Center, Los Angeles, CA USA; 289 The Hebrew University Faculty of Medicine, Jerusalem, Israel; 290 Barts Cancer Institute, Barts and the London School of Medicine and Dentistry, Queen Mary University of London, London, UK; 291 Department of Computer Science, Bioinformatics Group, University of Leipzig, Leipzig, Germany; 292 Interdisciplinary Center for Bioinformatics, University of Leipzig, Leipzig, Germany; 293 Transcriptome Bioinformatics, LIFE Research Center for Civilization Diseases, University of Leipzig, Leipzig, Germany; 294 Department of Medical Oncology, Dana-Farber Cancer Institute, Boston, MA USA; 295 Department of Cancer Biology, Dana-Farber Cancer Institute, Boston, MA USA; 296 Harvard Medical School, Boston, MA USA; 297 USC Norris Comprehensive Cancer Center, University of Southern California, Los Angeles, CA USA; 298 Department of Diagnostics and Public Health, University and Hospital Trust of Verona, Verona, Italy; 299 Department of Mathematics, Aarhus University, Aarhus, Denmark; 300 Department of Molecular Medicine (MOMA), Aarhus University Hospital, Aarhus N, Denmark; 301 Instituto Carlos Slim de la Salud, Mexico City, Mexico; 302 Department of Medical Biophysics, University of Toronto, Toronto, ON Canada; 303 Cancer Division, Garvan Institute of Medical Research, Kinghorn Cancer Centre, University of New South Wales (UNSW Sydney), Sydney, NSW Australia; 304 South Western Sydney Clinical School, Faculty of Medicine, University of New South Wales (UNSW Sydney), Liverpool, NSW Australia; 305 West of Scotland Pancreatic Unit, Glasgow Royal Infirmary, Glasgow, UK; 306 Center for Digital Health, Berlin Institute of Health and Charitè - Universitätsmedizin Berlin, Berlin, Germany; 307 Heidelberg Center for Personalized Oncology (DKFZ-HIPO), German Cancer Research Center (DKFZ), Heidelberg, Germany; 308 The Preston Robert Tisch Brain Tumor Center, Duke University Medical Center, Durham, NC USA; 309 Massachusetts General Hospital, Boston, MA USA; 310 National Institute of Biomedical Genomics, Kalyani, West Bengal India; 311 Institute of Clinical Medicine and Institute of Oral Biology, University of Oslo, Oslo, Norway; 312 University of North Carolina at Chapel Hill, Chapel Hill, NC USA; 313 ARC-Net Centre for Applied Research on Cancer, University and Hospital Trust of Verona, Verona, Italy; 314 The Institute of Cancer Research, London, UK; 315 Centre for Computational Biology, Duke-NUS Medical School, Singapore, Singapore; 316 Programme in Cancer and Stem Cell Biology, Duke-NUS Medical School, Singapore, Singapore; 317 Division of Oncology and Pathology, Department of Clinical Sciences Lund, Lund University, Lund, Sweden; 318 Department of Pediatric Oncology, Hematology and Clinical Immunology, Heinrich-Heine-University, Düsseldorf, Germany; 319 Laboratory for Medical Science Mathematics, RIKEN Center for Integrative Medical Sciences, Yokohama, Japan; 320 RIKEN Center for Integrative Medical Sciences, Yokohama, Japan; 321 Department of Internal Medicine/Hematology, Friedrich-Ebert-Hospital, Neumünster, Germany; 322 Departments of Dermatology and Pathology, Yale University, New Haven, CT USA; 323 Centre for Genomic Regulation (CRG), The Barcelona Institute of Science and Technology, Barcelona, Spain; 324 Radcliffe Department of Medicine, University of Oxford, Oxford, UK; 325 Canadian Center for Computational Genomics, McGill University, Montreal, QC Canada; 326 Department of Human Genetics, McGill University, Montreal, QC Canada; 327 Department of Human Genetics, University of California Los Angeles, Los Angeles, CA USA; 328 Department of Pharmacology, University of Toronto, Toronto, ON Canada; 329 Faculty of Medicine and Health Technology, Tampere University and Tays Cancer Center, Tampere University Hospital, Tampere, Finland; 330 Haematology, Leeds Teaching Hospitals NHS Trust, Leeds, UK; 331 Translational Research and Innovation, Centre Léon Bérard, Lyon, France; 332 Fox Chase Cancer Center, Philadelphia, PA USA; 333 International Agency for Research on Cancer, World Health Organization, Lyon, France; 334 Earlham Institute, Norwich, UK; 335 Norwich Medical School, University of East Anglia, Norwich, UK; 336 Department of Molecular Biology, Faculty of Science, Radboud Institute for Molecular Life Sciences, Radboud University, Nijmegen, HB The Netherlands; 337 CRUK Manchester Institute and Centre, Manchester, UK; 338 Department of Radiation Oncology, University of Toronto, Toronto, ON Canada; 339 Division of Cancer Sciences, Manchester Cancer Research Centre, University of Manchester, Manchester, UK; 340 Radiation Medicine Program, Princess Margaret Cancer Centre, Toronto, ON Canada; 341 Department of Pathology, Brigham and Women’s Hospital, Harvard Medical School, Boston, MA USA; 342 Department of Surgery, Division of Thoracic Surgery, The Johns Hopkins University School of Medicine, Baltimore, MD USA; 343 Division of Molecular Pathology, The Netherlands Cancer Institute, Oncode Institute, Amsterdam, CX The Netherlands; 344 Department of Biomolecular Engineering, University of California Santa Cruz, Santa Cruz, CA USA; 345 UC Santa Cruz Genomics Institute, University of California Santa Cruz, Santa Cruz, CA USA; 346 Division of Applied Bioinformatics, German Cancer Research Center (DKFZ), Heidelberg, Germany; 347 German Cancer Consortium (DKTK), German Cancer Research Center (DKFZ), Heidelberg, Germany; 348 National Center for Tumor Diseases (NCT) Heidelberg, Heidelberg, Germany; 349 Center for Biological Sequence Analysis, Department of Bio and Health Informatics, Technical University of Denmark, Lyngby, Denmark; 350 Novo Nordisk Foundation Center for Protein Research, University of Copenhagen, Copenhagen, Denmark; 351 Institute for Molecular Bioscience, University of Queensland, St. Lucia, Brisbane, QLD Australia; 352 Biomedical Engineering, Oregon Health and Science University, Portland, OR USA; 353 Division of Theoretical Bioinformatics, German Cancer Research Center (DKFZ), Heidelberg, Germany; 354 Institute of Pharmacy and Molecular Biotechnology and BioQuant, Heidelberg University, Heidelberg, Germany; 355 Federal Ministry of Education and Research, Berlin, Germany; 356 Melanoma Institute Australia, University of Sydney, Sydney, NSW Australia; 357 Pediatric Hematology and Oncology, University Hospital Muenster, Muenster, Germany; 358 Department of Pathology, Johns Hopkins University School of Medicine, Baltimore, MD USA; 359 McKusick-Nathans Institute of Genetic Medicine, Sidney Kimmel Comprehensive Cancer Center at Johns Hopkins University School of Medicine, Baltimore, MD USA; 360 Foundation Medicine, Inc, Cambridge, MA USA; 361 Department of Biomedical Data Science, Stanford University School of Medicine, Stanford, CA USA; 362 Department of Genetics, Stanford University School of Medicine, Stanford, CA USA; 363 Bakar Computational Health Sciences Institute and Department of Pediatrics, University of California, San Francisco, CA USA; 364 Institute of Clinical Medicine, Faculty of Medicine, University of Oslo, Oslo, Norway; 365 National Cancer Institute, National Institutes of Health, Bethesda, MD USA; 366 Royal Marsden NHS Foundation Trust, London and Sutton, UK; 367 Genome Biology Unit, European Molecular Biology Laboratory (EMBL), Heidelberg, Germany; 368 Department of Oncology, University of Cambridge, Cambridge, UK; 369 Li Ka Shing Centre, Cancer Research UK Cambridge Institute, University of Cambridge, Cambridge, UK; 370 Institut Gustave Roussy, Villejuif, France; 371 Cambridge University Hospitals NHS Foundation Trust, Cambridge, UK; 372 Department of Haematology, University of Cambridge, Cambridge, UK; 373 Anatomia Patológica, Hospital Clinic, Institut d’Investigacions Biomèdiques August Pi i Sunyer (IDIBAPS), University of Barcelona, Barcelona, Spain; 374 Spanish Ministry of Science and Innovation, Madrid, Spain; 375 University of Michigan Comprehensive Cancer Center, Ann Arbor, MI USA; 376 Department for BioMedical Research, University of Bern, Bern, Switzerland; 377 Department of Medical Oncology, Inselspital, University Hospital and University of Bern, Bern, Switzerland; 378 Graduate School for Cellular and Biomedical Sciences, University of Bern, Bern, Switzerland; 379 University of Pavia, Pavia, Italy; 380 University of Alabama at Birmingham, Birmingham, AL USA; 381 UHN Program in BioSpecimen Sciences, Toronto General Hospital, Toronto, ON Canada; 382 Department of Urology, Icahn School of Medicine at Mount Sinai, New York, NY USA; 383 Centre for Law and Genetics, University of Tasmania, Sandy Bay Campus, Hobart, TAS Australia; 384 Faculty of Biosciences, Heidelberg University, Heidelberg, Germany; 385 Department of Biochemistry, Microbiology and Immunology, Faculty of Medicine, University of Ottawa, Ottawa, ON Canada; 386 Division of Anatomic Pathology, Mayo Clinic, Rochester, MN USA; 387 Division of Cancer Epidemiology and Genetics, National Cancer Institute, National Institutes of Health, Bethesda, MD USA; 388 Illawarra Shoalhaven Local Health District L3 Illawarra Cancer Care Centre, Wollongong Hospital, Wollongong, NSW Australia; 389 BioForA, French National Institute for Agriculture, Food, and Environment (INRAE), ONF, Orléans, France; 390 Department of Biostatistics, Bloomberg School of Public Health, Johns Hopkins University, Baltimore, MD USA; 391 University of California San Diego, San Diego, CA USA; 392 Division of Experimental Pathology, Mayo Clinic, Rochester, MN USA; 393 Centre for Cancer Research, The Westmead Institute for Medical Research, University of Sydney, Sydney, NSW Australia; 394 Department of Gynaecological Oncology, Westmead Hospital, Sydney, NSW Australia; 395 PDXen Biosystems Inc, Seoul, South Korea; 396 Korea Advanced Institute of Science and Technology, Daejeon, South Korea; 397 Electronics and Telecommunications Research Institute, Daejeon, South Korea; 398 Institut National du Cancer (INCA), Boulogne-Billancourt, France; 399 Department of Genetics, Informatics Institute, University of Alabama at Birmingham, Birmingham, AL USA; 400 Division of Medical Oncology, National Cancer Centre, Singapore, Singapore; 401 Medical Oncology, University and Hospital Trust of Verona, Verona, Italy; 402 Department of Pediatrics, University Hospital Schleswig-Holstein, Kiel, Germany; 403 Hepatobiliary/Pancreatic Surgical Oncology Program, University Health Network, Toronto, ON Canada; 404 School of Biological Sciences, University of Auckland, Auckland, New Zealand; 405 Department of Surgery, University of Melbourne, Parkville, VIC Australia; 406 The Murdoch Children’s Research Institute, Royal Children’s Hospital, Parkville, VIC Australia; 407 Walter and Eliza Hall Institute, Parkville, VIC Australia; 408 Vancouver Prostate Centre, Vancouver, Canada; 409 Lunenfeld-Tanenbaum Research Institute, Mount Sinai Hospital, Toronto, ON Canada; 410 University of East Anglia, Norwich, UK; 411 Norfolk and Norwich University Hospital NHS Trust, Norwich, UK; 412 Victorian Institute of Forensic Medicine, Southbank, VIC Australia; 413 Department of Biomedical Informatics, Harvard Medical School, Boston, MA USA; 414 Department of Chemistry, Centre for Molecular Science Informatics, University of Cambridge, Cambridge, UK; 415 Ludwig Center at Harvard Medical School, Boston, MA USA; 416 Human Genome Sequencing Center, Baylor College of Medicine, Houston, TX USA; 417 Peter MacCallum Cancer Centre, University of Melbourne, Melbourne, VIC Australia; 418 Physics Division, Optimization and Systems Biology Lab, Massachusetts General Hospital, Boston, MA USA; 419 Department of Medicine, Baylor College of Medicine, Houston, TX USA; 420 University of Cologne, Cologne, Germany; 421 International Genomics Consortium, Phoenix, AZ USA; 422 Genomics Research Program, Ontario Institute for Cancer Research, Toronto, ON Canada; 423 Barking Havering and Redbridge University Hospitals NHS Trust, Romford, UK; 424 Children’s Hospital at Westmead, University of Sydney, Sydney, NSW Australia; 425 Department of Medicine, Section of Endocrinology, University and Hospital Trust of Verona, Verona, Italy; 426 Computational Biology Center, Memorial Sloan Kettering Cancer Center, New York, NY USA; 427 Department of Biology, ETH Zurich, Zürich, Switzerland; 428 Department of Computer Science, ETH Zurich, Zurich, Switzerland; 429 SIB Swiss Institute of Bioinformatics, Lausanne, Switzerland; 430 Weill Cornell Medical College, New York, NY USA; 431 Academic Department of Medical Genetics, University of Cambridge, Addenbrooke’s Hospital, Cambridge, UK; 432 MRC Cancer Unit, University of Cambridge, Cambridge, UK; 433 Departments of Pediatrics and Genetics, University of North Carolina at Chapel Hill, Chapel Hill, NC USA; 434 Seven Bridges Genomics, Charlestown, MA USA; 435 Annai Systems, Inc, Carlsbad, CA USA; 436 Department of Pathology, General Hospital of Treviso, Department of Medicine, University of Padua, Treviso, Italy; 437 Department of Computational Biology, University of Lausanne, Lausanne, Switzerland; 438 Department of Genetic Medicine and Development, University of Geneva Medical School, Geneva, CH Switzerland; 439 Swiss Institute of Bioinformatics, University of Geneva, Geneva, CH Switzerland; 440 The Francis Crick Institute, London, UK; 441 University of Leuven, Leuven, Belgium; 442 Institute of Medical Genetics and Applied Genomics, University of Tübingen, Tübingen, Germany; 443 Computational and Systems Biology, Genome Institute of Singapore, Singapore, Singapore; 444 School of Computing, National University of Singapore, Singapore, Singapore; 445 Big Data Institute, Li Ka Shing Centre, University of Oxford, Oxford, UK; 446 Biomedical Data Science Laboratory, Francis Crick Institute, London, UK; 447 Bioinformatics Group, Department of Computer Science, University College London, London, UK; 448 The Edward S. Rogers Sr. Department of Electrical and Computer Engineering, University of Toronto, Toronto, ON Canada; 449 Breast Cancer Translational Research Laboratory JC Heuson, Institut Jules Bordet, Brussels, Belgium; 450 Department of Oncology, Laboratory for Translational Breast Cancer Research, KU Leuven, Leuven, Belgium; 451 Institute for Research in Biomedicine (IRB Barcelona), The Barcelona Institute of Science and Technology, Barcelona, Spain; 452 Research Program on Biomedical Informatics, Universitat Pompeu Fabra, Barcelona, Spain; 453 Division of Medical Oncology, Princess Margaret Cancer Centre, Toronto, ON Canada; 454 Department of Physiology and Biophysics, Weill Cornell Medicine, New York, NY USA; 455 Institute for Computational Biomedicine, Weill Cornell Medicine, New York, NY USA; 456 Department of Pathology, UPMC Shadyside, Pittsburgh, PA USA; 457 Independent Consultant, Wellesley, USA; 458 Department of Cell and Molecular Biology, Science for Life Laboratory, Uppsala University, Uppsala, Sweden; 459 Department of Medicine and Department of Genetics, Washington University School of Medicine, St. Louis, St. Louis, MO USA; 460 Hefei University of Technology, Anhui, China; 461 Translational Cancer Research Unit, GZA Hospitals St.-Augustinus, Center for Oncological Research, Faculty of Medicine and Health Sciences, University of Antwerp, Antwerp, Belgium; 462 Simon Fraser University, Burnaby, BC Canada; 463 University of Pennsylvania, Philadelphia, PA USA; 464 Faculty of Science and Technology, University of Vic—Central University of Catalonia (UVic-UCC), Vic, Spain; 465 The Wellcome Trust, London, UK; 466 The Hospital for Sick Children, Toronto, ON Canada; 467 Department of Pathology, Queen Elizabeth University Hospital, Glasgow, UK; 468 Department of Genetics and Computational Biology, QIMR Berghofer Medical Research Institute, Brisbane, QLD Australia; 469 Department of Oncology, Centre for Cancer Genetic Epidemiology, University of Cambridge, Cambridge, UK; 470 Department of Public Health and Primary Care, Centre for Cancer Genetic Epidemiology, University of Cambridge, Cambridge, UK; 471 Prostate Cancer Canada, Toronto, ON Canada; 472 University of Cambridge, Cambridge, UK; 473 Department of Laboratory Medicine, Translational Cancer Research, Lund University Cancer Center at Medicon Village, Lund University, Lund, Sweden; 474 Heidelberg University, Heidelberg, Germany; 475 New BIH Digital Health Center, Berlin Institute of Health (BIH) and Charité - Universitätsmedizin Berlin, Berlin, Germany; 476 CIBER Epidemiología y Salud Pública (CIBERESP), Madrid, Spain; 477 Research Group on Statistics, Econometrics and Health (GRECS), UdG, Barcelona, Spain; 478 Quantitative Genomics Laboratories (qGenomics), Barcelona, Spain; 479 Icelandic Cancer Registry, Icelandic Cancer Society, Reykjavik, Iceland; 480 State Key Laboratory of Cancer Biology, and Xijing Hospital of Digestive Diseases, Fourth Military Medical University, Shaanxi, China; 481 Department of Medicine (DIMED), Surgical Pathology Unit, University of Padua, Padua, Italy; 482 Rigshospitalet, Copenhagen, Denmark; 483 Center for Cancer Genomics, National Cancer Institute, National Institutes of Health, Bethesda, MD USA; 484 Department of Biochemistry and Molecular Medicine, University of Montreal, Montreal, QC Canada; 485 Australian Institute of Tropical Health and Medicine, James Cook University, Douglas, QLD Australia; 486 Department of Neuro-Oncology, Istituto Neurologico Besta, Milano, Italy; 487 Bioplatforms Australia, North Ryde, NSW Australia; 488 Department of Pathology (Research), University College London Cancer Institute, London, UK; 489 Department of Surgical Oncology, Princess Margaret Cancer Centre, Toronto, ON Canada; 490 Department of Medical Oncology, Josephine Nefkens Institute and Cancer Genomics Centre, Erasmus Medical Center, Rotterdam, CN The Netherlands; 491 The University of Queensland Thoracic Research Centre, The Prince Charles Hospital, Brisbane, QLD Australia; 492 CIBIO/InBIO - Research Center in Biodiversity and Genetic Resources, Universidade do Porto, Vairão, Portugal; 493 HCA Laboratories, London, UK; 494 University of Liverpool, Liverpool, UK; 495 The Azrieli Faculty of Medicine, Bar-Ilan University, Safed, Israel; 496 Department of Neurosurgery, University of Florida, Gainesville, FL USA; 497 Department of Pathology, Graduate School of Medicine, University of Tokyo, Tokyo, Japan; 498 University of Milano Bicocca, Monza, Italy; 499 BGI-Shenzhen, Shenzhen, China; 500 Department of Pathology, Oslo University Hospital Ulleval, Oslo, Norway; 501 Center for Biomedical Informatics, Harvard Medical School, Boston, MA USA; 502 Department Biochemistry and Molecular Biomedicine, University of Barcelona, Barcelona, Spain; 503 Office of Cancer Genomics, National Cancer Institute, National Institutes of Health, Bethesda, MD USA; 504 Cancer Epigenomics, German Cancer Research Center (DKFZ), Heidelberg, Germany; 505 Department of Cancer Biology, The University of Texas MD Anderson Cancer Center, Houston, TX USA; 506 Department of Surgical Oncology, The University of Texas MD Anderson Cancer Center, Houston, TX USA; 507 Department of Computer Science, Yale University, New Haven, CT USA; 508 Department of Molecular Biophysics and Biochemistry, Yale University, New Haven, CT USA; 509 Program in Computational Biology and Bioinformatics, Yale University, New Haven, CT USA; 510 Center for Cancer Research, Massachusetts General Hospital, Boston, MA USA; 511 Department of Pathology, Massachusetts General Hospital, Boston, MA USA; 512 Department of Pathology, Memorial Sloan Kettering Cancer Center, New York, NY USA; 513 Division of Gastroenterology and Hepatology, Mayo Clinic, Rochester, MN USA; 514 University of Sydney, Sydney, NSW Australia; 515 University of Oxford, Oxford, UK; 516 Department of Surgery, Academic Urology Group, University of Cambridge, Cambridge, UK; 517 Department of Medicine II, University of Würzburg, Wuerzburg, Germany; 518 Sylvester Comprehensive Cancer Center, University of Miami, Miami, FL USA; 519 Institut Hospital del Mar d’Investigacions Mèdiques (IMIM), Barcelona, Spain; 520 Genome Integrity and Structural Biology Laboratory, National Institute of Environmental Health Sciences (NIEHS), Durham, NC USA; 521 St. Thomas’s Hospital, London, UK; 522 Osaka International Cancer Center, Osaka, Japan; 523 Department of Pathology, Skåne University Hospital, Lund University, Lund, Sweden; 524 Department of Medical Oncology, Beatson West of Scotland Cancer Centre, Glasgow, UK; 525 National Human Genome Research Institute, National Institutes of Health, Bethesda, MD USA; 526 Centre for Cancer Research, Victorian Comprehensive Cancer Centre, University of Melbourne, Melbourne, VIC Australia; 527 Department of Medicine, Section of Hematology/Oncology, University of Chicago, Chicago, IL USA; 528 German Center for Infection Research (DZIF), Partner Site Hamburg-Borstel-Lübeck-Riems, Hamburg, Germany; 529 Bioinformatics Research Centre (BiRC), Aarhus University, Aarhus, Denmark; 530 Department of Biotechnology, Ministry of Science and Technology, Government of India, New Delhi, Delhi India; 531 National Cancer Centre Singapore, Singapore, Singapore; 532 Brandeis University, Waltham, MA USA; 533 Department of Urologic Sciences, University of British Columbia, Vancouver, BC Canada; 534 Department of Internal Medicine, Stanford University, Stanford, CA USA; 535 The University of Texas Health Science Center at Houston, Houston, TX USA; 536 Imperial College NHS Trust, Imperial College, London, INY UK; 537 Senckenberg Institute of Pathology, University of Frankfurt Medical School, Frankfurt, Germany; 538 Department of Medicine, Division of Biomedical Informatics, UC San Diego School of Medicine, San Diego, CA USA; 539 Center for Precision Health, School of Biomedical Informatics, The University of Texas Health Science Center, Houston, TX USA; 540 Oxford Nanopore Technologies, New York, NY USA; 541 Institute of Medical Science, University of Tokyo, Tokyo, Japan; 542 Howard Hughes Medical Institute, University of California Santa Cruz, Santa Cruz, CA USA; 543 Wakayama Medical University, Wakayama, Japan; 544 Department of Internal Medicine, Division of Medical Oncology, Lineberger Comprehensive Cancer Center, University of North Carolina at Chapel Hill, Chapel Hill, NC USA; 545 University of Tennessee Health Science Center for Cancer Research, Memphis, TN USA; 546 Department of Histopathology, Salford Royal NHS Foundation Trust, Salford, UK; 547 Faculty of Biology, Medicine and Health, University of Manchester, Manchester, UK; 548 BIOPIC, ICG and College of Life Sciences, Peking University, Beijing, China; 549 Peking-Tsinghua Center for Life Sciences, Peking University, Beijing, China; 550 Children’s Hospital of Philadelphia, Philadelphia, PA USA; 551 Department of Bioinformatics and Computational Biology and Department of Systems Biology, The University of Texas MD Anderson Cancer Center, Houston, TX USA; 552 Karolinska Institute, Stockholm, Sweden; 553 The Donnelly Centre, University of Toronto, Toronto, ON Canada; 554 Department of Medical Genetics, College of Medicine, Hallym University, Chuncheon, South Korea; 555 Department of Experimental and Health Sciences, Institute of Evolutionary Biology (UPF-CSIC), Universitat Pompeu Fabra, Barcelona, Spain; 556 Health Data Science Unit, University Clinics, Heidelberg, Germany; 557 Massachusetts General Hospital Center for Cancer Research, Charlestown, MA USA; 558 Hokkaido University, Sapporo, Japan; 559 Department of Pathology and Clinical Laboratory, National Cancer Center Hospital, Tokyo, Japan; 560 Department of Genetics, University of North Carolina at Chapel Hill, Chapel Hill, NC USA; 561 Computational Biology, Leibniz Institute on Aging - Fritz Lipmann Institute (FLI), Jena, Germany; 562 University of Melbourne Centre for Cancer Research, Melbourne, VIC Australia; 563 University of Nebraska Medical Center, Omaha, NE USA; 564 Syntekabio Inc, Daejeon, South Korea; 565 Department of Pathology, Academic Medical Center, Amsterdam, AZ The Netherlands; 566 China National GeneBank-Shenzhen, Shenzhen, China; 567 Division of Molecular Genetics, German Cancer Research Center (DKFZ), Heidelberg, Germany; 568 Division of Life Science and Applied Genomics Center, Hong Kong University of Science and Technology, Clear Water Bay, Hong Kong, China; 569 Icahn School of Medicine at Mount Sinai, New York, NY USA; 570 Geneplus-Shenzhen, Shenzhen, China; 571 School of Computer Science and Technology, Xi’an Jiaotong University, Xi’an, China; 572 AbbVie, North Chicago, IL USA; 573 Institute of Pathology, Charité – University Medicine Berlin, Berlin, Germany; 574 Centre for Translational and Applied Genomics, British Columbia Cancer Agency, Vancouver, BC Canada; 575 Edinburgh Royal Infirmary, Edinburgh, UK; 576 Berlin Institute for Medical Systems Biology, Max Delbrück Center for Molecular Medicine, Berlin, Germany; 577 Department of Pediatric Immunology, Hematology and Oncology, University Hospital, Heidelberg, Germany; 578 German Cancer Research Center (DKFZ), Heidelberg, Germany; 579 Heidelberg Institute for Stem Cell Technology and Experimental Medicine (HI-STEM), Heidelberg, Germany; 580 Institute for Computational Biomedicine, Weill Cornell Medical College, New York, NY USA; 581 New York Genome Center, New York, NY USA; 582 Department of Urology, James Buchanan Brady Urological Institute, Johns Hopkins University School of Medicine, Baltimore, MD USA; 583 Department of Preventive Medicine, Graduate School of Medicine, The University of Tokyo, Tokyo, Japan; 584 Department of Molecular and Cellular Biology, Baylor College of Medicine, Houston, TX USA; 585 Department of Pathology and Immunology, Baylor College of Medicine, Houston, TX USA; 586 Michael E. DeBakey Veterans Affairs Medical Center, Houston, TX USA; 587 Technical University of Denmark, Lyngby, Denmark; 588 Department of Pathology, College of Medicine, Hanyang University, Seoul, South Korea; 589 Academic Unit of Surgery, School of Medicine, College of Medical, Veterinary and Life Sciences, University of Glasgow, Glasgow Royal Infirmary, Glasgow, UK; 590 Department of Pathology, Asan Medical Center, College of Medicine, Ulsan University, Songpa-gu, Seoul South Korea; 591 Science Writer, Garrett Park, MD USA; 592 International Cancer Genome Consortium (ICGC)/ICGC Accelerating Research in Genomic Oncology (ARGO) Secretariat, Ontario Institute for Cancer Research, Toronto, ON Canada; 593 University of Ljubljana, Ljubljana, Slovenia; 594 Department of Public Health Sciences, University of Chicago, Chicago, IL USA; 595 Research Institute, NorthShore University HealthSystem, Evanston, IL USA; 596 Department for Biomedical Research, University of Bern, Bern, Switzerland; 597 Centre of Genomics and Policy, McGill University and Génome Québec Innovation Centre, Montreal, QC Canada; 598 Carolina Center for Genome Sciences, University of North Carolina at Chapel Hill, Chapel Hill, NC USA; 599 Hopp Children’s Cancer Center (KiTZ), Heidelberg, Germany; 600 Pediatric Glioma Research Group, German Cancer Research Center (DKFZ), Heidelberg, Germany; 601 Cancer Research UK, London, UK; 602 Indivumed GmbH, Hamburg, Germany; 603 Genome Integration Data Center, Syntekabio, Inc, Daejeon, South Korea; 604 University Hospital Zurich, Zurich, Switzerland; 605 Clinical Bioinformatics, Swiss Institute of Bioinformatics, Geneva, Switzerland; 606 Institute for Pathology and Molecular Pathology, University Hospital Zurich, Zurich, Switzerland; 607 Institute of Molecular Life Sciences, University of Zurich, Zurich, Switzerland; 608 MRC Human Genetics Unit, MRC IGMM, University of Edinburgh, Edinburgh, UK; 609 Women’s Cancer Program at the Samuel Oschin Comprehensive Cancer Institute, Cedars-Sinai Medical Center, Los Angeles, CA USA; 610 Department of Biology, Bioinformatics Group, Division of Molecular Biology, Faculty of Science, University of Zagreb, Zagreb, Croatia; 611 Department for Internal Medicine II, University Hospital Schleswig-Holstein, Kiel, Germany; 612 Genetics and Molecular Pathology, SA Pathology, Adelaide, SA Australia; 613 Department of Gastric Surgery, National Cancer Center Hospital, Tokyo, Japan; 614 Department of Bioinformatics, Division of Cancer Genomics, National Cancer Center Research Institute, Tokyo, Japan; 615 A.A. Kharkevich Institute of Information Transmission Problems, Moscow, Russia; 616 Oncology and Immunology, Dmitry Rogachev National Research Center of Pediatric Hematology, Moscow, Russia; 617 Skolkovo Institute of Science and Technology, Moscow, Russia; 618 Department of Surgery, The George Washington University, School of Medicine and Health Science, Washington, DC USA; 619 Endocrine Oncology Branch, Center for Cancer Research, National Cancer Institute, National Institutes of Health, Bethesda, MD USA; 620 Melanoma Institute Australia, Macquarie University, Sydney, NSW Australia; 621 MIT Computer Science and Artificial Intelligence Laboratory, Massachusetts Institute of Technology, Cambridge, MA USA; 622 Tissue Pathology and Diagnostic Oncology, Royal Prince Alfred Hospital, Sydney, NSW Australia; 623 Cholangiocarcinoma Screening and Care Program and Liver Fluke and Cholangiocarcinoma Research Centre, Faculty of Medicine, Khon Kaen University, Khon Kaen, Thailand; 624 Controlled Department and Institution, New York, NY USA; 625 Englander Institute for Precision Medicine, Weill Cornell Medicine, New York, NY USA; 626 National Cancer Center, Gyeonggi, South Korea; 627 Department of Biochemistry, College of Medicine, Ewha Womans University, Seoul, South Korea; 628 Health Sciences Department of Biomedical Informatics, University of California San Diego, La Jolla, CA USA; 629 Research Core Center, National Cancer Centre Korea, Goyang-si, South Korea; 630 Department of Health Sciences and Technology, Sungkyunkwan University School of Medicine, Seoul, South Korea; 631 Samsung Genome Institute, Seoul, South Korea; 632 Breast Oncology Program, Dana-Farber/Brigham and Women’s Cancer Center, Boston, MA USA; 633 Department of Surgery, Memorial Sloan Kettering Cancer Center, New York, NY USA; 634 Division of Breast Surgery, Brigham and Women’s Hospital, Boston, MA USA; 635 Integrative Bioinformatics Support Group, National Institute of Environmental Health Sciences (NIEHS), Durham, NC USA; 636 Department of Clinical Science, University of Bergen, Bergen, Norway; 637 Center For Medical Innovation, Seoul National University Hospital, Seoul, South Korea; 638 Department of Internal Medicine, Seoul National University Hospital, Seoul, South Korea; 639 Institute of Computer Science, Polish Academy of Sciences, Warsawa, Poland; 640 Functional and Structural Genomics, German Cancer Research Center (DKFZ), Heidelberg, Germany; 641 Laboratory of Translational Genomics, Division of Cancer Epidemiology and Genetics, National Cancer Institute, , National Institutes of Health, Bethesda, MD USA; 642 Institute for Medical Informatics Statistics and Epidemiology, University of Leipzig, Leipzig, Germany; 643 Morgan Welch Inflammatory Breast Cancer Research Program and Clinic, The University of Texas MD Anderson Cancer Center, Houston, TX USA; 644 Department of Hematology and Oncology, Georg-Augusts-University of Göttingen, Göttingen, Germany; 645 Institute of Cell Biology (Cancer Research), University of Duisburg-Essen, Essen, Germany; 646 King’s College London and Guy’s and St. Thomas’ NHS Foundation Trust, London, UK; 647 Center for Epigenetics, Van Andel Research Institute, Grand Rapids, MI USA; 648 The University of Queensland Centre for Clinical Research, Royal Brisbane and Women’s Hospital, Herston, QLD Australia; 649 Department of Pediatric Oncology and Hematology, University of Cologne, Cologne, Germany; 650 University of Düsseldorf, Düsseldorf, Germany; 651 Department of Pathology, Institut Jules Bordet, Brussels, Belgium; 652 Institute of Biomedicine, Sahlgrenska Academy at University of Gothenburg, Gothenburg, Sweden; 653 Children’s Medical Research Institute, Sydney, NSW Australia; 654 ILSbio, LLC Biobank, Chestertown, MD USA; 655 Division of Genetics and Genomics, Boston Children’s Hospital, Harvard Medical School, Boston, MA USA; 656 Institute for Bioengineering and Biopharmaceutical Research (IBBR), Hanyang University, Seoul, South Korea; 657 Department of Statistics, University of California Santa Cruz, Santa Cruz, CA USA; 658 National Genotyping Center, Institute of Biomedical Sciences, Academia Sinica, Taipei, Taiwan; 659 Department of Vertebrate Genomics/Otto Warburg Laboratory Gene Regulation and Systems Biology of Cancer, Max Planck Institute for Molecular Genetics, Berlin, Germany; 660 McGill University and Genome Quebec Innovation Centre, Montreal, QC Canada; 661 biobyte solutions GmbH, Heidelberg, Germany; 662 Gynecologic Oncology, NYU Laura and Isaac Perlmutter Cancer Center, New York University, New York, NY USA; 663 Division of Oncology, Stem Cell Biology Section, Washington University School of Medicine, St. Louis, MO USA; 664 Department of Systems Biology, The University of Texas MD Anderson Cancer Center, Houston, TX USA; 665 Harvard University, Cambridge, MA USA; 666 Urologic Oncology Branch, Center for Cancer Research, National Cancer Institute, National Institutes of Health, Bethesda, MD USA; 667 University of Oslo, Oslo, Norway; 668 University of Toronto, Toronto, ON Canada; 669 Peking University, Beijing, China; 670 School of Life Sciences, Peking University, Beijing, China; 671 Leidos Biomedical Research, Inc, McLean, VA USA; 672 Hematology, Hospital Clinic, Institut d’Investigacions Biomèdiques August Pi i Sunyer (IDIBAPS), University of Barcelona, Barcelona, Spain; 673 Second Military Medical University, Shanghai, China; 674 Chinese Cancer Genome Consortium, Shenzhen, China; 675 Department of Medical Oncology, Beijing Hospital, Beijing, China; 676 Laboratory of Molecular Oncology, Key Laboratory of Carcinogenesis and Translational Research (Ministry of Education), Peking University Cancer Hospital and Institute, Beijing, China; 677 School of Medicine/School of Mathematics and Statistics, University of St. Andrews, St, Andrews, Fife UK; 678 Institute for Systems Biology, Seattle, WA USA; 679 Department of Biochemistry and Molecular Biology, Faculty of Medicine, University Institute of Oncology-IUOPA, Oviedo, Spain; 680 Institut Bergonié, Bordeaux, France; 681 Cancer Unit, MRC University of Cambridge, Cambridge, UK; 682 Department of Pathology and Laboratory Medicine, Center for Personalized Medicine, Children’s Hospital Los Angeles, Los Angeles, CA USA; 683 John Curtin School of Medical Research, Canberra, ACT Australia; 684 MVZ Department of Oncology, PraxisClinic am Johannisplatz, Leipzig, Germany; 685 Department of Information Technology, Ghent University, Ghent, Belgium; 686 Department of Plant Biotechnology and Bioinformatics, Ghent University, Ghent, Belgium; 687 Institute for Genomic Medicine, Nationwide Children’s Hospital, Columbus, OH USA; 688 Computational Biology Program, School of Medicine, Oregon Health and Science University, Portland, OR USA; 689 Department of Surgery, Duke University, Durham, NC USA; 690 Institució Catalana de Recerca i Estudis Avançats (ICREA), Barcelona, Spain; 691 Institut Català de Paleontologia Miquel Crusafont, Universitat Autònoma de Barcelona, Barcelona, Spain; 692 University of Glasgow, Glasgow, UK; 693 Institut d’Investigacions Biomèdiques August Pi i Sunyer (IDIBAPS), Barcelona, Spain; 694 Division of Oncology, Washington University School of Medicine, St. Louis, MO USA; 695 Department of Surgery and Cancer, Imperial College, London, INY UK; 696 Applications Department, Oxford Nanopore Technologies, Oxford, UK; 697 Department of Obstetrics, Gynecology and Reproductive Services, University of California San Francisco, San Francisco, CA USA; 698 Department of Biochemistry and Molecular Medicine, University California at Davis, Sacramento, CA USA; 699 STTARR Innovation Facility, Princess Margaret Cancer Centre, Toronto, ON Canada; 700 Discipline of Surgery, Western Sydney University, Penrith, NSW Australia; 701 Yale School of Medicine, Yale University, New Haven, CT USA; 702 Department of Genetics, Lineberger Comprehensive Cancer Center, University of North Carolina at Chapel Hill, Chapel Hill, NC USA; 703 Departments of Neurology and Neurosurgery, Henry Ford Hospital, Detroit, MI USA; 704 Precision Oncology, OHSU Knight Cancer Institute, Oregon Health and Science University, Portland, OR USA; 705 Institute of Pathology, University Medical Center Hamburg-Eppendorf, Hamburg, Germany; 706 Department of Health Sciences, Faculty of Medical Sciences, Kyushu University, Fukuoka, Japan; 707 Heidelberg Academy of Sciences and Humanities, Heidelberg, Germany; 708 Department of Clinical Pathology, University of Melbourne, Melbourne, VIC, Australia; 709 Department of Pathology, Roswell Park Cancer Institute, Buffalo, NY USA; 710 Department of Computer Science, University of Helsinki, Helsinki, Finland; 711 Institute of Biotechnology, University of Helsinki, Helsinki, Finland; 712 Organismal and Evolutionary Biology Research Programme, University of Helsinki, Helsinki, Finland; 713 Department of Obstetrics and Gynecology, Division of Gynecologic Oncology, Washington University School of Medicine, St. Louis, MO USA; 714 Penrose St. Francis Health Services, Colorado Springs, CO USA; 715 Institute of Pathology, Ulm University and University Hospital of Ulm, Ulm, Germany; 716 National Cancer Center, Tokyo, Japan; 717 Genome Institute of Singapore, Singapore, Singapore; 718 32Program in Computational Biology and Bioinformatics, Yale University, New Haven, CT USA; 719 German Cancer Aid, Bonn, Germany; 720 Programme in Cancer and Stem Cell Biology, Centre for Computational Biology, Duke-NUS Medical School, Singapore, Singapore; 721 The Chinese University of Hong Kong, Shatin, NT, Hong Kong China; 722 Fourth Military Medical University, Shaanxi, China; 723 The University of Cambridge School of Clinical Medicine, Cambridge, UK; 724 St. Jude Children’s Research Hospital, Memphis, TN USA; 725 University Health Network, Princess Margaret Cancer Centre, Toronto, ON Canada; 726 Center for Biomolecular Science and Engineering, University of California Santa Cruz, Santa Cruz, CA USA; 727 Department of Medicine, University of Chicago, Chicago, IL USA; 728 Department of Neurology, Mayo Clinic, Rochester, MN USA; 729 Cambridge Oesophagogastric Centre, Cambridge University Hospitals NHS Foundation Trust, Cambridge, UK; 730 Department of Computer Science, Carleton College, Northfield, MN USA; 731 Institute of Cancer Sciences, College of Medical Veterinary and Life Sciences, University of Glasgow, Glasgow, UK; 732 Department of Epidemiology, University of Alabama at Birmingham, Birmingham, AL USA; 733 HudsonAlpha Institute for Biotechnology, Huntsville, AL USA; 734 O’Neal Comprehensive Cancer Center, University of Alabama at Birmingham, Birmingham, AL USA; 735 Department of Pathology, Keio University School of Medicine, Tokyo, Japan; 736 Department of Hepatobiliary and Pancreatic Oncology, National Cancer Center Hospital, Tokyo, Japan; 737 Sage Bionetworks, Seattle, WA USA; 738 Lymphoma Genomic Translational Research Laboratory, National Cancer Centre, Singapore, Singapore; 739 Department of Clinical Pathology, Robert-Bosch-Hospital, Stuttgart, Germany; 740 Department of Cell and Systems Biology, University of Toronto, Toronto, ON Canada; 741 Department of Biosciences and Nutrition, Karolinska Institutet, Stockholm, Sweden; 742 Center for Liver Cancer, Research Institute and Hospital, National Cancer Center, Gyeonggi, South Korea; 743 Division of Hematology-Oncology, Samsung Medical Center, Sungkyunkwan University School of Medicine, Seoul, South Korea; 744 Samsung Advanced Institute for Health Sciences and Technology, Sungkyunkwan University School of Medicine, Seoul, South Korea; 745 Cheonan Industry-Academic Collaboration Foundation, Sangmyung University, Cheonan, South Korea; 746 NYU Langone Medical Center, New York, NY USA; 747 Department of Hematology and Medical Oncology, Cleveland Clinic, Cleveland, OH USA; 748 Department of Radiation Oncology, University of California San Francisco, San Francisco, CA USA; 749 Department of Health Sciences Research, Mayo Clinic, Rochester, MN USA; 750 Helen F. Graham Cancer Center at Christiana Care Health Systems, Newark, DE USA; 751 Heidelberg University Hospital, Heidelberg, Germany; 752 CSRA Incorporated, Fairfax, VA USA; 753 Research Department of Pathology, University College London Cancer Institute, London, UK; 754 Department of Research Oncology, Guy’s Hospital, King’s Health Partners AHSC, King’s College London School of Medicine, London, UK; 755 Faculty of Medicine and Health Sciences, Macquarie University, Sydney, NSW Australia; 756 University Hospital of Minjoz, INSERM UMR 1098, Besançon, France; 757 Spanish National Cancer Research Centre, Madrid, Spain; 758 Center of Digestive Diseases and Liver Transplantation, Fundeni Clinical Institute, Bucharest, Romania; 759 Cureline, Inc, South San Francisco, CA USA; 760 St. Luke’s Cancer Centre, Royal Surrey County Hospital NHS Foundation Trust, Guildford, UK; 761 Cambridge Breast Unit, Addenbrooke’s Hospital, Cambridge University Hospital NHS Foundation Trust and NIHR Cambridge Biomedical Research Centre, Cambridge, UK; 762 East of Scotland Breast Service, Ninewells Hospital, Aberdeen, UK; 763 Department of Genetics, Microbiology and Statistics, University of Barcelona, IRSJD, IBUB, Barcelona, Spain; 764 Department of Obstetrics and Gynecology, Medical College of Wisconsin, Milwaukee, WI USA; 765 Hematology and Medical Oncology, Winship Cancer Institute of Emory University, Atlanta, GA USA; 766 Department of Computer Science, Princeton University, Princeton, NJ USA; 767 Vanderbilt Ingram Cancer Center, Vanderbilt University, Nashville, TN USA; 768 Ohio State University College of Medicine and Arthur G. James Comprehensive Cancer Center, Columbus, OH USA; 769 Department of Surgery, Yokohama City University Graduate School of Medicine, Kanagawa, Japan; 770 Division of Chromatin Networks, German Cancer Research Center (DKFZ) and BioQuant, Heidelberg, Germany; 771 Research Computing Center, University of North Carolina at Chapel Hill, Chapel Hill, NC USA; 772 School of Molecular Biosciences and Center for Reproductive Biology, Washington State University, Pullman, WA USA; 773 Finsen Laboratory and Biotech Research and Innovation Centre (BRIC), University of Copenhagen, Copenhagen, Denmark; 774 Department of Laboratory Medicine and Pathobiology, University of Toronto, Toronto, ON Canada; 775 Department of Pathology, Human Oncology and Pathogenesis Program, Memorial Sloan Kettering Cancer Center, New York, NY USA; 776 University Hospital Giessen, Pediatric Hematology and Oncology, Giessen, Germany; 777 Oncologie Sénologie, ICM Institut Régional du Cancer, Montpellier, France; 778 Institute of Clinical Molecular Biology, Christian-Albrechts-University, Kiel, Germany; 779 Institute of Pathology, University of Wuerzburg, Wuerzburg, Germany; 780 Department of Urology, North Bristol NHS Trust, Bristol, UK; 781 SingHealth, Duke-NUS Institute of Precision Medicine, National Heart Centre Singapore, Singapore, Singapore; 782 Department of Computer Science, University of Toronto, Toronto, ON Canada; 783 Bern Center for Precision Medicine, University Hospital of Bern, University of Bern, Bern, Switzerland; 784 Englander Institute for Precision Medicine, Weill Cornell Medicine and New York Presbyterian Hospital, New York, NY USA; 785 Meyer Cancer Center, Weill Cornell Medicine, New York, NY USA; 786 Pathology and Laboratory, Weill Cornell Medical College, New York, NY USA; 787 Vall d’Hebron Institute of Oncology: VHIO, Barcelona, Spain; 788 General and Hepatobiliary-Biliary Surgery, Pancreas Institute, University and Hospital Trust of Verona, Verona, Italy; 789 National Centre for Biological Sciences, Tata Institute of Fundamental Research, Bangalore, India; 790 Indiana University, Bloomington, IN USA; 791 Department of Pathology, GZA-ZNA Hospitals, Antwerp, Belgium; 792 Analytical Biological Services, Inc, Wilmington, DE USA; 793 Sydney Medical School, University of Sydney, Sydney, NSW Australia; 794 cBio Center, Dana-Farber Cancer Institute, Harvard Medical School, Boston, MA USA; 795 Department of Cell Biology, Harvard Medical School, Boston, MA USA; 796 Advanced Centre for Treatment Research and Education in Cancer, Tata Memorial Centre, Navi Mumbai, Maharashtra India; 797 School of Environmental and Life Sciences, Faculty of Science, The University of Newcastle, Ourimbah, NSW Australia; 798 Department of Dermatology, University Hospital of Essen, Essen, Germany; 799 Bioinformatics and Omics Data Analytics, German Cancer Research Center (DKFZ), Heidelberg, Germany; 800 Department of Urology, Charité Universitätsmedizin Berlin, Berlin, Germany; 801 Martini-Clinic, Prostate Cancer Center, University Medical Center Hamburg-Eppendorf, Hamburg, Germany; 802 Department of General Internal Medicine, University of Kiel, Kiel, Germany; 803 German Cancer Consortium (DKTK), Partner site Berlin, Berlin, Germany; 804 Cancer Research Institute, Beth Israel Deaconess Medical Center, Boston, MA USA; 805 University of Pittsburgh, Pittsburgh, PA USA; 806 Department of Ophthalmology and Ocular Genomics Institute, Massachusetts Eye and Ear, Harvard Medical School, Boston, MA USA; 807 Center for Psychiatric Genetics, NorthShore University HealthSystem, Evanston, IL USA; 808 Van Andel Research Institute, Grand Rapids, MI USA; 809 Laboratory of Molecular Medicine, Human Genome Center, Institute of Medical Science, University of Tokyo, Tokyo, Japan; 810 Japan Agency for Medical Research and Development, Tokyo, Japan; 811 Korea University, Seoul, South Korea; 812 Murtha Cancer Center, Walter Reed National Military Medical Center, Bethesda, MD USA; 813 Human Genetics, University of Kiel, Kiel, Germany; 814 Department of Oncologic Pathology, Dana-Farber Cancer Institute, Harvard Medical School, Boston, MA USA; 815 Oregon Health and Science University, Portland, OR USA; 816 Center for RNA Interference and Noncoding RNA, The University of Texas MD Anderson Cancer Center, Houston, TX USA; 817 Department of Experimental Therapeutics, The University of Texas MD Anderson Cancer Center, Houston, TX USA; 818 Department of Gynecologic Oncology and Reproductive Medicine, The University of Texas MD Anderson Cancer Center, Houston, TX USA; 819 University Hospitals Coventry and Warwickshire NHS Trust, Coventry, UK; 820 Department of Radiation Oncology, Radboud University Nijmegen Medical Centre, Nijmegen, GA The Netherlands; 821 Institute for Genomics and Systems Biology, University of Chicago, Chicago, IL USA; 822 Clinic for Hematology and Oncology, St.-Antonius-Hospital, Eschweiler, Germany; 823 Computational and Systems Biology Program, Memorial Sloan Kettering Cancer Center, New York, NY USA; 824 University of Iceland, Reykjavik, Iceland; 825 Division of Computational Genomics and Systems Genetics, German Cancer Research Center (DKFZ), Heidelberg, Germany; 826 Dundee Cancer Centre, Ninewells Hospital, Dundee, UK; 827 Department for Internal Medicine III, University of Ulm and University Hospital of Ulm, Ulm, Germany; 828 Institut Curie, INSERM Unit 830, Paris, France; 829 Department of Gastroenterology and Hepatology, Yokohama City University Graduate School of Medicine, Kanagawa, Japan; 830 Department of Laboratory Medicine, Radboud University Nijmegen Medical Centre, Nijmegen, GA The Netherlands; 831 Division of Cancer Genome Research, German Cancer Research Center (DKFZ), Heidelberg, Germany; 832 Department of General Surgery, Singapore General Hospital, Singapore, Singapore; 833 Cancer Science Institute of Singapore, National University of Singapore, Singapore, Singapore; 834 Department of Medical and Clinical Genetics, Genome-Scale Biology Research Program, University of Helsinki, Helsinki, Finland; 835 East Anglian Medical Genetics Service, Cambridge University Hospitals NHS Foundation Trust, Cambridge, UK; 836 Irving Institute for Cancer Dynamics, Columbia University, New York, NY USA; 837 Institute of Molecular and Cell Biology, Singapore, Singapore; 838 Laboratory of Cancer Epigenome, Division of Medical Science, National Cancer Centre Singapore, Singapore, Singapore; 839 Universite Lyon, INCa-Synergie, Centre Léon Bérard, Lyon, France; 840 Department of Urology, Mayo Clinic, Rochester, MN USA; 841 Royal National Orthopaedic Hospital - Stanmore, Stanmore, Middlesex UK; 842 Department of Biochemistry, Genetics and Immunology, University of Vigo, Vigo, Spain; 843 Giovanni Paolo II / I.R.C.C.S. Cancer Institute, Bari, BA Italy; 844 Neuroblastoma Genomics, German Cancer Research Center (DKFZ), Heidelberg, Germany; 845 Fondazione Policlinico Universitario Gemelli IRCCS, Rome, Italy, Rome, Italy; 846 University of Verona, Verona, Italy; 847 Centre National de Génotypage, CEA - Institute de Génomique, Evry, France; 848 CAPHRI Research School, Maastricht University, Maastricht, ER The Netherlands; 849 Department of Biopathology, Centre Léon Bérard, Lyon, France; 850 Université Claude Bernard Lyon 1, Villeurbanne, France; 851 Core Research for Evolutional Science and Technology (CREST), JST, Tokyo, Japan; 852 Department of Biological Sciences, Laboratory for Medical Science Mathematics, Graduate School of Science, University of Tokyo, Yokohama, Japan; 853 Department of Medical Science Mathematics, Medical Research Institute, Tokyo Medical and Dental University (TMDU), Tokyo, Japan; 854 Cancer Ageing and Somatic Mutation Programme, Wellcome Sanger Institute, Hinxton, UK; 855 University Hospitals Birmingham NHS Foundation Trust, Birmingham, UK; 856 Centre for Cancer Research and Cell Biology, Queen’s University, Belfast, UK; 857 Breast Medical Oncology, The University of Texas MD Anderson Cancer Center, Houston, TX USA; 858 Department of Surgery, Johns Hopkins University School of Medicine, Baltimore, MD USA; 859 Department of Oncology-Pathology, Science for Life Laboratory, Karolinska Institute, Stockholm, Sweden; 860 School of Cancer Sciences, Faculty of Medicine, University of Southampton, Southampton, UK; 861 Department of Gene Technology, Tallinn University of Technology, Tallinn, Estonia; 862 Genetics and Genome Biology Program, SickKids Research Institute, The Hospital for Sick Children, Toronto, ON Canada; 863 Departments of Neurosurgery and Hematology and Medical Oncology, Winship Cancer Institute and School of Medicine, Emory University, Atlanta, GA USA; 864 Department of Clinical and Molecular Medicine, Faculty of Medicine and Health Sciences, Norwegian University of Science and Technology, Trondheim, Norway; 865 Argmix Consulting, North Vancouver, BC Canada; 866 Department of Information Technology, Ghent University, Interuniversitair Micro-Electronica Centrum (IMEC), Ghent, Belgium; 867 Nuffield Department of Surgical Sciences, John Radcliffe Hospital, University of Oxford, Oxford, UK; 868 Institute of Mathematics and Computer Science, University of Latvia, Riga, LV Latvia; 869 Discipline of Pathology, Sydney Medical School, University of Sydney, Sydney, NSW Australia; 870 Department of Applied Mathematics and Theoretical Physics, Centre for Mathematical Sciences, University of Cambridge, Cambridge, UK; 871 Department of Epidemiology and Biostatistics, Memorial Sloan Kettering Cancer Center, New York, NY USA; 872 Department of Statistics, Columbia University, New York, NY USA; 873 Department of Immunology, Genetics and Pathology, Science for Life Laboratory, Uppsala University, Uppsala, Sweden; 874 School of Electronic and Information Engineering, Xi’an Jiaotong University, Xi’an, China; 875 Department of Histopathology, Cambridge University Hospitals NHS Foundation Trust, Cambridge, UK; 876 Oxford NIHR Biomedical Research Centre, University of Oxford, Oxford, UK; 877 Georgia Regents University Cancer Center, Augusta, GA USA; 878 Wythenshawe Hospital, Manchester, UK; 879 Department of Genetics, Washington University School of Medicine, St.Louis, MO USA; 880 Department of Biological Oceanography, Leibniz Institute of Baltic Sea Research, Rostock, Germany; 881 Wellcome Centre for Human Genetics, University of Oxford, Oxford, UK; 882 Department of Molecular and Human Genetics, Baylor College of Medicine, Houston, TX USA; 883 Thoracic Oncology Laboratory, Mayo Clinic, Rochester, MN USA; 884 Institute for Genomic Medicine, Nationwide Children’s Hospital, Columbus, OH USA; 885 Department of Obstetrics and Gynecology, Division of Gynecologic Oncology, Mayo Clinic, Rochester, MN USA; 886 International Institute for Molecular Oncology, Poznań, Poland; 887 Poznan University of Medical Sciences, Poznań, Poland; 888 Genomics and Proteomics Core Facility High Throughput Sequencing Unit, German Cancer Research Center (DKFZ), Heidelberg, Germany; 889 NCCS-VARI Translational Research Laboratory, National Cancer Centre Singapore, Singapore, Singapore; 890 Edison Family Center for Genome Sciences and Systems Biology, Washington University, St. Louis, MO USA; 891 MRC-University of Glasgow Centre for Virus Research, Glasgow, UK; 892 Department of Medical Informatics and Clinical Epidemiology, Division of Bioinformatics and Computational Biology, OHSU Knight Cancer Institute, Oregon Health and Science University, Portland, OR USA; 893 School of Electronic Information and Communications, Huazhong University of Science and Technology, Wuhan, China; 894 Department of Applied Mathematics and Statistics, Johns Hopkins University, Baltimore, MD USA; 895 Department of Cancer Genome Informatics, Graduate School of Medicine, Osaka University, Osaka, Japan; 896 Institute of Computer Science, Heidelberg University, Heidelberg, Germany; 897 School of Mathematics and Statistics, University of Sydney, Sydney, NSW Australia; 898 Ben May Department for Cancer Research, University of Chicago, Chicago, IL USA; 899 Department of Human Genetics, University of Chicago, Chicago, IL USA; 900 Tri-Institutional PhD Program in Computational Biology and Medicine, Weill Cornell Medicine, New York, NY USA; 901 The First Affiliated Hospital, Xi’an Jiaotong University, Xi’an, China; 902 Department of Medicine and Therapeutics, The Chinese University of Hong Kong, Shatin, NT, Hong Kong China; 903 Department of Biostatistics, The University of Texas MD Anderson Cancer Center, Houston, TX USA; 904 Duke-NUS Medical School, Singapore, Singapore; 905 Department of Surgery, Ruijin Hospital, Shanghai Jiaotong University School of Medicine, Shanghai, China; 906 School of Computing Science, University of Glasgow, Glasgow, UK; 907 Division of Orthopaedic Surgery, Oslo University Hospital, Oslo, Norway; 908 Eastern Clinical School, Monash University, Melbourne, VIC Australia; 909 Epworth HealthCare, Richmond, VIC Australia; 910 Department of Biostatistics and Computational Biology, Dana-Farber Cancer Institute and Harvard Medical School, Boston, MA USA; 911 Department of Biomedical Informatics, College of Medicine, The Ohio State University, Columbus, OH USA; 912 The Ohio State University Comprehensive Cancer Center (OSUCCC – James), Columbus, OH USA; 913 The University of Texas School of Biomedical Informatics (SBMI) at Houston, Houston, TX USA; 914 Department of Biostatistics, University of North Carolina at Chapel Hill, Chapel Hill, NC USA; 915 Department of Biochemistry and Molecular Genetics, Feinberg School of Medicine, Northwestern University, Chicago, IL USA; 916 Faculty of Medicine and Health, University of Sydney, Sydney, NSW Australia; 917 Department of Pathology, Erasmus Medical Center Rotterdam, Rotterdam, GD The Netherlands; 918 Division of Molecular Carcinogenesis, The Netherlands Cancer Institute, Amsterdam, CX The Netherlands; 919 Institute of Molecular Life Sciences and Swiss Institute of Bioinformatics, University of Zurich, Zurich, Switzerland

**Keywords:** Cancer, Genomics

## Abstract

About half of all cancers have somatic integrations of retrotransposons. Here, to characterize their role in oncogenesis, we analyzed the patterns and mechanisms of somatic retrotransposition in 2,954 cancer genomes from 38 histological cancer subtypes within the framework of the Pan-Cancer Analysis of Whole Genomes (PCAWG) project. We identified 19,166 somatically acquired retrotransposition events, which affected 35% of samples and spanned a range of event types. Long interspersed nuclear element (LINE-1; L1 hereafter) insertions emerged as the first most frequent type of somatic structural variation in esophageal adenocarcinoma, and the second most frequent in head-and-neck and colorectal cancers. Aberrant L1 integrations can delete megabase-scale regions of a chromosome, which sometimes leads to the removal of tumor-suppressor genes, and can induce complex translocations and large-scale duplications. Somatic retrotranspositions can also initiate breakage–fusion–bridge cycles, leading to high-level amplification of oncogenes. These observations illuminate a relevant role of 22 L1 retrotransposition in remodeling the cancer genome, with potential implications for the development of human tumors.

## Abstract (web summary)

An analysis of 2,954 genomes from 38 cancer subtypes identified 19,166 retrotransposition events in 35% of samples. Aberrant LINE-1 retrotranspositions can lead to the deletion of tumor-suppressor genes as well as the amplification of oncogenes.

## Main

L1 retrotransposons are widespread repetitive elements in the human genome, representing 17% of the entire DNA content^1,2^. Using a combination of cellular enzymes and self-encoded proteins with endonuclease and reverse transcriptase activity, L1 elements copy and insert themselves at new genomic sites, in a process called retrotransposition. Most of the approximately 500,000 L1 copies in the human reference genome are truncated, inactive elements that are unable to retrotranspose. A small subset of them, around 100–150 L1 loci, remain active in the average human genome, acting as source elements, a small number of which consists of highly active copies termed hot-L1s^3–5^. These L1 source elements are usually transcriptionally repressed, but epigenetic changes that occur in tumors may promote their expression and allow them to retrotranspose^6,7^. Somatic L1 retrotransposition usually introduces a new copy of the 3′ end of the L1 sequence, and can also mobilize unique DNA sequences located immediately downstream of the source element, in a process called 3′ transduction^7–9^. L1 retrotransposons can also promote the somatic trans-mobilization of Alu elements, SINE-VNTR-Alu (SVA) elements and processed pseudogenes, which are copies of mRNAs that have been reverse transcribed into DNA and inserted into the genome with the machinery of active L1 elements^10–12^.

Approximately 50% of human tumors contain somatic retrotranspositions of L1 elements^7,13–15^. Previous analyses indicate that although a fraction of somatically acquired L1 insertions in cancer may influence gene function, the majority of retrotransposon integrations in a single tumor represent passenger mutations with little or no effect on cancer development^7,13^. Nonetheless, L1 elements are capable of promoting other types of genomic structural alterations in the germline and somatically, in addition to canonical L1 insertion events^16–18^; the effect of these alterations remains largely unexplored in the context of human cancer^19,20^.

To further understand the roles of retrotransposons in cancer, we developed strategies to analyze the patterns and mechanisms of somatic retrotransposition in 2,954 cancer genomes from 38 histological cancer subtypes within the framework of the PCAWG project^21^, many of which had not been evaluated for retrotransposition. On the basis of the robustness of the retrotransposition calls, we retained 296 tumors that were preliminarily excluded by the PCAWG Consortium^21^ (see Methods). Our analyses identify patterns and mutational mechanisms of structural variation in human cancers that are mediated by L1 retrotransposition. We found that the aberrant integration of L1 retrotransposons has a relevant role in remodeling the architecture of the cancer genome in some human tumors, mainly by promoting megabase-scale deletions that, occasionally, generate genomic consequences that may promote cancer development through the removal of tumor-suppressor genes, such as *CDKN2A*, or trigger the amplification of oncogenes, such as *CCND1*.

## Results

### The landscape of somatic retrotransposition in a large cancer whole-genome dataset

**Fig. 1:**
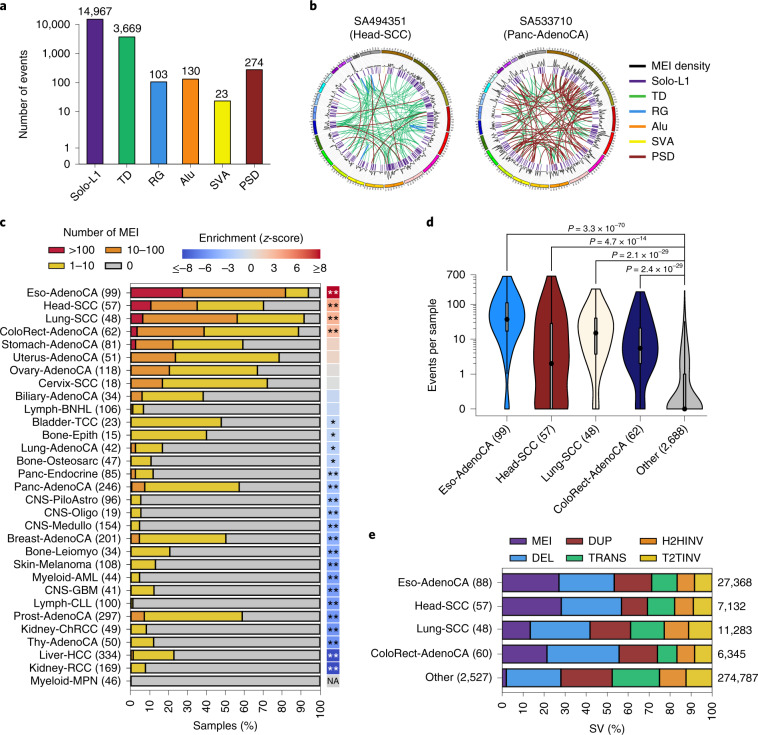
Landscape of somatic retrotransposition across human cancers. **a**) Number of somatic retrotransposition events identified in 2,954 cancer genomes across six categories: solo-L1, L1-mediated transductions (TD), L1-mediated rearrangements (RG), Alu, SVA and pseudogenes (PSD). **b**, Left, circos plot showing a head-and-neck tumor (Head-SCC) with high retrotransposition rate (638 somatic events). Right, a single pancreatic adenocarcinoma sample harboring around 26% (70 out of 274) of all processed pseudogenes identified in the PCAWG cohort. Chromosome ideograms are shown around the outer ring with individual rearrangements represented as arcs; colors match the type of rearrangement. **c**, For 31 PCAWG cancer types with sample size of *n* ≥ 15, data show the proportion of tumor samples with >100 (red), 10–100 (orange), 1–10 (yellow) and 0 (gray) somatic retrotranspositions. The number of samples analyzed for each tumor type is shown in parentheses. Retrotransposition enrichment or depletion for each tumor type together with the level of significance (zero-inflated negative binomial regression) is shown. *P < 0.05, **P < 0.01. NA, not applicable. **d**, Distribution of retrotransposition events per sample across the four tumor types significantly enriched in somatic retrotranspositions; the remaining tumors are grouped into ‘Other’. The number of samples from each group is shown in parentheses; point, median; box, 25th to 75th percentiles (interquartile range); whiskers, data within 1.5× the interquartile range. *P* values indicate significance from a two-tailed Mann–Whitney *U*-test. The *y* axis is shown on a logarithmic scale. **e**, For the same four tumor types in **d**, the fraction of structural variants (SV) belonging to six classes is shown: mobile element insertions (MEI), deletions (DEL), duplications (DUP), translocations (TRANS), head-to-head inversions (H2HINV) and tail-to-tail inversions (T2TINV). The total number of structural variants per cancer type is indicated on the right side of the panel.

We ran our bioinformatic pipelines (Methods and Supplementary Note) to explore somatic retrotransposition on whole-genome sequencing data from 2,954 tumors and their matched normal pairs, across 38 cancer types (Supplementary Fig. 1 and Supplementary Table 1). The analysis retrieved a total of 19,166 somatically acquired retrotranspositions that were classified into six categories (Fig. 1a and Supplementary Table 2). Comprising 98% (18,739 out of 19,166) of the events, L1 integrations (14,967 solo-L1, 3,669 L1-transductions, and 103 L1-mediated rearrangements, which mainly comprised deletions) overwhelmingly dominate the landscape of somatic retrotransposition in the PCAWG dataset (Fig. 1a,b). By contrast, elements of the lineages Alu (Supplementary Fig. 2) and SVA (comprising 130 and 23 somatic copies, respectively) and processed pseudogenes, with 274 events, represent minor categories.

The core pipeline, TraFiC-mem (Supplementary Fig. 3)—which was used to explore somatic retrotransposition in PCAWG—was validated by single-molecule whole-genome sequencing data analysis of one cancer cell line with high retrotransposition rate and its matched normal sample, confirming the somatic acquisition of 295 out of 308 retrotransposition events (false discovery rate <5%, Supplementary Fig. 4a,b). To further evaluate TraFiC-mem, we reanalyzed a mock cancer genome into which we had previously^7^ seeded somatic retrotransposition events at different levels of tumor clonality, and then simulated sequencing reads to the average level of coverage of the PCAWG dataset. The results confirmed a high precision (>99%) of TraFiC-mem, and a recall ranging from 90 to 94% for tumor clonalities from 25 to 100%, respectively (Supplementary Fig. 4c–e).

We observed marked variation in the retrotransposition rate across PCAWG tumor types (Fig. 1c and Supplementary Table 3). Overall, 35% (1,046 out of 2,954) of all cancer genomes have at least one retrotransposition event. However, esophageal adenocarcinoma, head-and-neck squamous carcinoma, lung squamous carcinoma and colorectal adenocarcinoma are significantly enriched in somatic retrotranspositions (Mann–Whitney *U*-test, *P* < 0.05; Fig. 1c,d and Supplementary Fig. 5). These four tumor types alone account for 70% (13,373 out of 19,166) of all somatic events in the PCAWG dataset, although they represent just 9% (266 out of 2,954) of the samples. This is particularly noticeable in esophageal adenocarcinoma, in which 27% (27 out of 99) of the samples show more than 100 separate somatic retrotranspositions (Fig. 1c), making L1 insertions the most frequent type of structural variation in esophageal adenocarcinoma (Fig. 1e). Furthermore, retrotranspositions are the second-most frequent type of structural variants in head-and-neck squamous and colorectal adenocarcinomas (Fig. 1e). To gain insights into the genetic causes that make some cancers more prone to retrotransposition than others, we looked for associations between retrotransposition and driver mutations in cancer-related genes. This analysis revealed an increased L1 retrotransposition rate in tumors with *TP53* mutations (Mann–Whitney *U*-test, *P* < 0.05; Supplementary Fig. 6), and supports previous analyses that have suggested that TP53 functions to restrain mobile elements^22,23^. We also observe a widespread correlation between L1 retrotransposition and other types of structural variation (Spearman’s *ρ* = 0.44, *P* < 0.01; Supplementary Fig. 7), a finding that is most likely a consequence of a confounding effect of *TP53*-mutated genotypes (Supplementary Fig. 6).

We identified 43% (7,979 out of 18,636) somatic retrotranspositions of L1 inserted within gene regions including promoters, of which 66 events hit cancer-associated genes. The analysis of expression levels in samples with available transcriptome data, revealed four genes—including the *ABL* oncogene—with L1 retrotranspositions in the proximity of promoter regions that showed significant overexpression compared with the expression in the remaining samples of the same tumor type (Student’s *t*-test, *q* < 0.10; Supplementary Fig. 8a–c). The structural analysis of RNA-sequencing data identified instances in which portions of a somatic retrotransposition within a gene exonize, a process that sometimes involves cancer-associated genes (Supplementary Fig. 8d). In addition, we found evidence of aberrant fusion transcripts arising from the inclusion of processed pseudogenes in the target host gene and expression of processed pseudogenes landing in intergenic regions (Supplementary Fig. 8e).

### Dissecting the genomic features that influence the landscape of L1 retrotranspositions in cancer

**Fig. 2:**
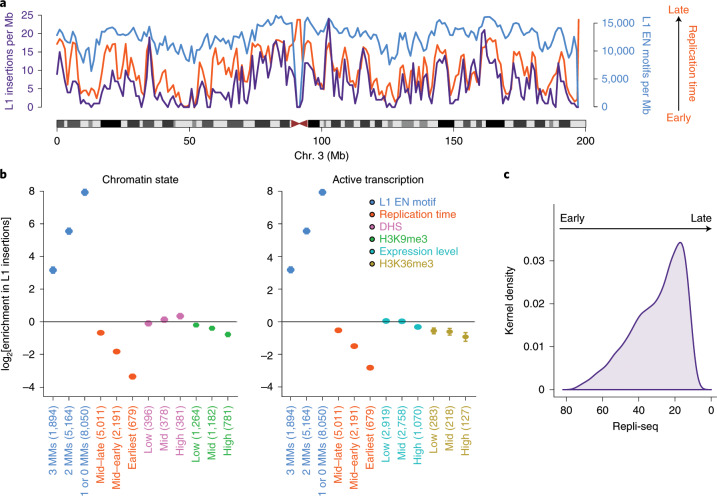
Distribution of L1 somatic insertions across the cancer genome and its association with genome organization features. Genome-wide analysis of the distribution of 15,906 somatic L1 insertions, which include solo-L1 and L1 transductions with a 3′-poly(A) breakpoint characterized to base-pair resolution. **a**, The L1 insertion rate (purple) is shown together with the L1 endonuclease (EN) motif density (blue) and replication timing (orange). The data are represented per 1-Mb window. For illustrative purposes, only chromosome 3 is shown. **b**, Association between L1 insertion rate and multiple predictor variables at single-nucleotide resolution. Enrichment scores (thick dots) are adjusted for multiple covariates and compare the L1 insertion rate in bins 1–3 for a particular genomic feature (L1 endonuclease motif, replication timing, open chromatin, histone marks and expression level) versus bin 0 of the same feature, which therefore always has log-transformed enrichment = 0 by definition and is not shown. The error bars represent 95% confidence intervals. The number of observations per bin is provided in parentheses. MMs, the number of mismatches with respect to the consensus L1 endonuclease motif (see Supplementary Note). Heterochromatic regions and transcription elongation are defined based on H3K9me3 and H3K36me3 histone marks. Accessible chromatin is measured through DNase hypersensitivity. **c**, L1 insertion density, using kernel density estimate (KDE), along the replication timing spectrum. DNA replication timing is expressed on a scale from 80 (early) to 0 (late).

The genome-wide analysis of the distribution of somatic L1 insertions across the cancer genome revealed considerable variation in the rate of L1 retrotransposition (Fig. 2a and Supplementary Table 4). To understand the reasons behind such variation, we studied the association of L1 event rates with various genomic features. We first investigated whether the distribution of somatic L1s across the cancer genome could be determined by the occurrence of L1-endonuclease target-site motifs. We used a statistical approach based on negative binomial regression to deconvolute the influence of multiple overlapping genomic variables^24^; this analysis showed that close matches to the motif have a 244-fold increased L1 rate, compared with non-matched motifs (Fig. 2b and Supplementary Fig. 9a). Adjusting for this effect, we found a strong association with DNA replication time; the latest-replicating quarter of the genome was 8.9-fold enriched in L1 events (95% confidence interval, 8.25–9.71) compared with the earliest-replicating quarter (Fig. 2b,c and Supplementary Fig. 9b). Recent work^25^ has shown that L1 retrotransposition has a strong cell-cycle bias, and preferentially occurs during S phase. Our results are in agreement with these findings and suggest that L1 retrotransposition peaks in the later stages of nuclear DNA synthesis.

Next, we examined L1 rates in open chromatin measured using DNase hypersensitivity and, conversely, in closed heterochromatic regions by analyzing K9-trimethylated histone H3 (H3K9me3)^26^. When adjusting for the confounding effects of L1 motif content and replication time^24^, we found that somatic L1 events are enriched in open chromatin (1.27-fold in the top DNase hypersensitivity bin; 95% confidence interval, 1.14–1.41; Fig. 2b) and depleted in heterochromatin (1.72-fold, 95% confidence interval, 1.57–1.99; Fig. 2b). This finding differs from previous analyses, which have suggested that L1 insertions favored heterochomatin^7^—a discrepancy that we believe to be due to the confounding effect between heterochromatin and late-replicating DNA regions, which was not addressed in previous analyses. We also found a negative association of L1 rate with features of active transcription of chromatin, characterized by fewer L1 events at active promoters (1.63-fold; Supplementary Fig. 9c), a slight but significant reduction in L1 rates in highly expressed genes (1.25-fold lower; 95% confidence interval, 1.16–1.34; Fig. 2b) and a further depletion at H3K36me3 (1.90-fold reduction in the highest tertile; 95% confidence interval, 1.59–2.29; Fig. 2b), a mark of actively transcribed regions deposited in the body and at the 3′ end of active genes^26^. Further details on these associations are shown in Supplementary Fig. 9c–e and described in the Supplementary Note.

### The contribution of L1 source elements to the pan-cancer retrotransposition burden

**Fig. 3:**
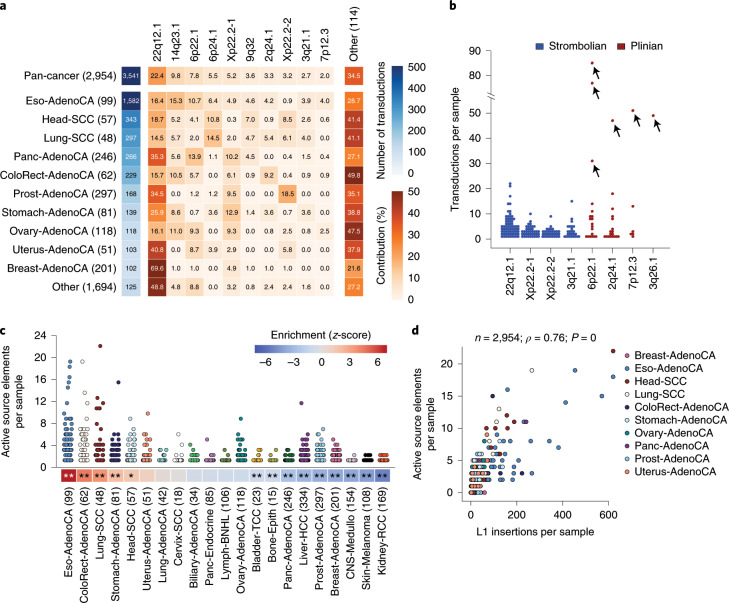
The dynamics of L1 source-element activity in human cancer. **a**, The total number of transductions identified for each cancer type is shown as a blue-colored scale. The sample size for each tumor type is shown in parentheses. Contribution of each source element is defined as the proportion of the total number of transductions from each cancer type that is explained by each source locus. Only the top ten contributing source elements are shown, while the remaining are grouped into the category ‘Other’. **b**, Two extreme patterns of hot-L1 activity, Strombolian (blue) and Plinian (red), were identified. Dots show the number of transductions promoted by each source element in a given tumor sample. Arrows highlight violent eruptions (that is, strong peaks of somatic activity) in particular samples. **c**, Number of active germline L1 source elements per sample, across cancer types with source element activity. A source element is considered to be active in a given sample if it promotes at least one transduction. The enrichment or depletion of the number of active source elements for each tumor type together with the level of significance (zero-inflated negative binomial regression) is shown. *P < 0.05, **P < 0.01. The number of samples analyzed for each tumor type is shown in parentheses. **d**, Correlation between the number of somatic L1 insertions and the number of active germline L1 source elements in PCAWG samples. Each dot represents a tumor sample and colors match cancer types. Sample sizes (*n*), together with Spearman’s *ρ* and *P* values are shown above the panel.

We used somatically mobilized L1 3′ transduction events to trace L1 activity to specific source elements^7^. This strategy revealed 124 germline L1 loci in the human genome that are responsible for most of the genomic variation generated by retrotransposition in the PCAWG dataset^7,21^ (Supplementary Table 5). To our knowledge, 52 of these loci represent previously unreported source elements in human cancer^21^. We analyzed the relative contribution of individual source elements to retrotransposition burden across cancer types, and found that retrotransposition is generally dominated by five hot-L1 source elements that alone give rise to half of all somatic transductions (Fig. 3a). This analysis revealed a dichotomous pattern of hot-L1 activity, with source elements that we have termed Strombolian and Plinian, given their similarity to these two types of volcanoes (Fig. 3b). Strombolian source elements are relatively indolent and produce small numbers of retrotranspositions in individual tumor samples, although they are often active and contribute substantially to overall retrotransposition in the PCAWG dataset. By contrast, Plinian elements are rarely active across tumors, but in these isolated cases, their activity is fulminant, causing large numbers of retrotranspositions.

At the individual tumor level, although we observed that the number of active source elements in a single cancer genome varied from 1 to 22, typically only 1 to 3 loci were operative (Fig. 3c). There is a correlation between somatic retrotranspositions and the number of active germline L1 source elements among PCAWG samples (Fig. 3d); this is likely one of the factors that explains why esophageal adenocarcinoma, lung and head-and-neck squamous carcinoma account for higher retrotransposition rates—in these three tumor types we also observed higher numbers of active germline L1 loci (Fig. 3c). Occasionally, somatic L1 integrations that retain their full length may also act as a source for subsequent somatic retrotransposition events^7,27^, and may reach high activity rates, leading them to dominate retrotransposition in a given tumor. For example, in a remarkable head-and-neck tumor sample, SA197656, we identified one somatic L1 integration at 4p16.1 that then triggered 18 transductions from its new site, with the next most active element being a germline L1 locus at 22q12.1, which accounted for 15 transductions (Supplementary Table 5).

### Genomic deletions mediated by somatic L1 retrotransposition

**Fig. 4:**
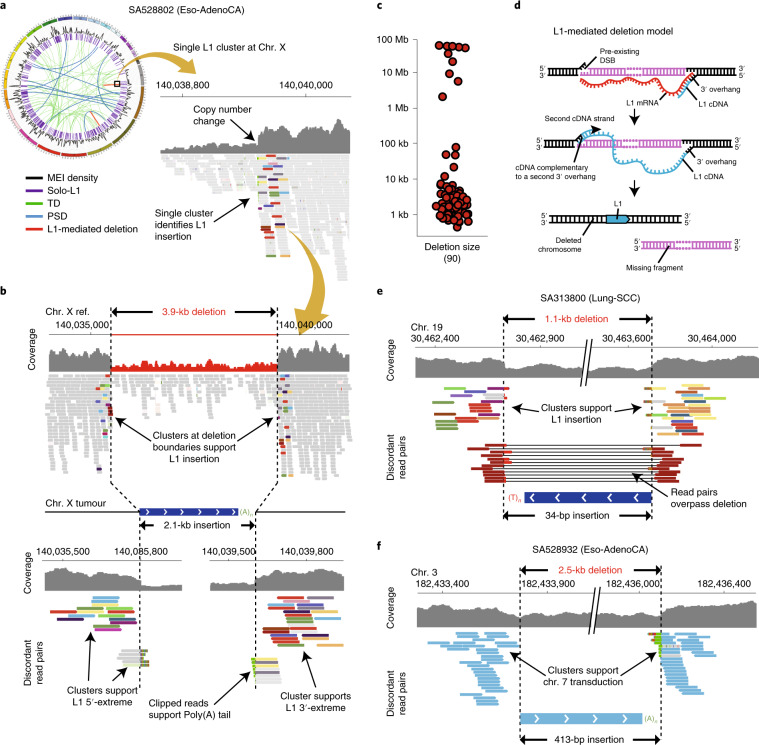
The hallmarks of somatic L1-mediated deletions revealed by copy-number and paired-end mapping analysis. **a**, In esophageal adenocarcinoma sample SA528802, we found a single cluster of reads on chromosome X, which is associated with one breakpoint of a copy-number loss, and for which the mates unequivocally identified one extreme of a somatic L1 integration. Paired-end reads are colored by the chromosome on which their mates can be found. Different colors for different reads from the same cluster indicate that mates are mapping a repetitive element. **b**, Analysis of the associated copy-number change on chromosome X identifies the missing L1 reciprocal cluster at the second breakpoint of the copy-number loss, and reveals a 3.9-kb deletion that occurs in conjunction with the integration of a 2.1-kb L1 somatic insertion. (A)_*n*_ and (T)_*n*_ represent poly(A) and poly(T) tails, respectively. **c**, Model of L1-mediated deletion. The integration of an L1 mRNA starts with L1-endonuclease cleavage promoting a 3′ overhang for reverse transcription. The cDNA (−) strand invades a second 3′ overhang from a pre-existing double-strand break upstream of the initial integration site. **d**, Distribution of the sizes of 90 L1-mediated deletions identified in the PCAWG dataset. **e**, In lung squamous carcinoma sample SA313800, a 34-bp truncated L1 insertion promotes a 1.1-kb deletion on chromosome 19. Because the L1 insertion was so short, we also identified discordant read pairs that span the L1 event and support the deletion. **f**, In esophageal adenocarcinoma sample SA528932, the integration on chromosome 3 of a 413-bp orphan L1 transduction from chromosome 7 causes a 2.5-kb deletion, which is supported by two clusters of discordant read pairs for which the mates map onto the transduced region of chromosome 7.

In cancer genomes with high somatic L1 activity rates, we observed that some L1 retrotransposition events followed a distinctive pattern that consisted of a single cluster of reads, associated with copy-number loss, for which the mates unequivocally identified one extreme of a somatic L1 integration with, apparently, no local, reciprocal cluster that supported the other extreme of the L1 insertion (Fig. 4a). Analysis of the associated copy-number changes identified the missing L1 reciprocal cluster at the far end of the copy-number loss, indicating that this pattern represents a deletion that occurred in conjunction with the integration of an L1 retrotransposon (Fig. 4b; see the Supplementary Note for additional information on how to interpret the paired-end mapping data from this and other figures). These rearrangements—called L1-mediated deletions—have been observed to occur somatically with engineered L1s in cultured human cells^16,17^ and naturally in the brain^18^, and are most likely the consequence of an aberrant mechanism of L1 integration.

We developed specific algorithms to systematically identify L1-mediated deletions, and applied these methods across all PCAWG tumors. We identified 90 somatic events that matched the patterns described above, causing deletions of different size, which ranged in size from around 0.5 kb to 53.4 Mb (Fig. 4c and Supplementary Table 6). The reconstruction of the sequence at the breakpoint junctions in each case supports the presence of an L1-element—or L1-transduction—sequence and its companion polyadenylate tract, indicative of passage through an RNA intermediate. No target site duplication was found, which is also the typical pattern for L1-mediated deletions^17^. One potential mechanism for these events is that a molecule of L1 cDNA pairs with a distant 3′ overhang from a pre-existing double-strand DNA break generated upstream of the initial integration site, and the DNA region between the break and the original target site is subsequently removed by aberrant repair^17^ (Fig. 4d). Indeed, in 75% (47 out of 63) of L1-mediated deletions with a 5′-end breakpoint characterized to base-pair resolution, the analysis of the sequences at the junction revealed short (1–5 bp long, with median at 3 bp) microhomologies between the pre-integration site and the 5′ L1 sequence integrated right there (Supplementary Table 6). Furthermore, we found 14% (9 out of 63) instances in which short insertions (1–33 bp long, with median at 9 bp) are found at the 5′-breakpoint junction of the insertion. Both signatures are consistent with a non-homologous end-joining mechanism^28^, or other type of microhomology-mediated repair, for the 5′-end attachment of the L1 cDNA to a 3′ overhang from a pre-existing double-strand DNA break located upstream. L1-mediated deletions in which microhomologies or insertions are not found may follow alternative models^17,29–31^.

To confirm that these rearrangements are mediated by the integration of a single intervening retrotransposition event, we explored the PCAWG dataset for somatic L1-mediated deletions in which the L1 sequences at both breakpoints of the deletion could unequivocally be assigned to the same L1 insertion. These include small deletions and associated L1 insertions that were shorter than the library size, allowing sequencing read pairs to overlay the entire structure. For example, in a lung tumor sample, SA313800, we identified a deletion involving a 1-kb region of 19q12 with hallmarks of being generated by an L1 element (Fig. 4e). In this rearrangement, we found two different types of discordant read pairs at the deletion breakpoints: one cluster that supported the insertion of an L1 element and a second that spanned the L1 event and supported the deletion. Another type of L1-mediated deletion that could unequivocally be assigned to a single L1 insertion event is represented by those deletions generated by the integration of orphan L1 transductions. These transductions represent fragments of unique DNA sequence located downstream of an active L1 locus, which are mobilized without the companion L1 (refs. ^7,15^). For example, in one esophageal tumor sample, SA528932, we found a deletion of 2.5 kb on chromosome 3 mediated by the orphan transduction of a sequence downstream of an L1 locus on chromosome 7 (Fig. 4f).

Owing to the unavailability of PCAWG DNA specimens, we performed a validation of 16 additional somatic L1-mediated deletions that were identified by TraFiC-mem in two head-and-neck cancer cell lines with high retrotransposition rates, NCI-H2009 and NCI-H2087^7^. We carried out two independent validation approaches, including PCR followed by single-molecule sequencing of amplicons, and Illumina whole-genome sequencing using mate-pair libraries with long insert size (3 kb and 10 kb). The results confirmed the somatic status of the rearrangements and a single L1-derived retrotransposition as the cause of the associated copy-number loss (Supplementary Figs. 10–12 and Supplementary Table 7).

Analysis of L1 3′-extreme insertion breakpoint sequences from L1-mediated deletions found in the PCAWG dataset revealed that 82% (74 out of 90) of the L1 events that caused deletions preferentially inserted into sequences that resemble L1-endonuclease consensus cleavage sites (for example, 5′-TTTT/A-3′ and related sequences^32^) (Supplementary Table 6). This confirms that the L1 machinery, through a target-primed reverse-transcription mechanism, is responsible for the integration of most of the L1 events that cause neighboring DNA loss^32^. Notably, in 16% (14 out of 90) of the events endonucleotidic cleavage occurred at the phosphodiester bond between a T and G instead of between the standard T and A site. In addition, we observed 8% (7 out of 90) instances in which the endonuclease motif was not found and the integrated element was truncated at both the 5′ and 3′ ends, suggesting that a small fraction of L1-associated deletions are the consequence of an L1-endonuclease-independent insertion mechanism^30–32^. Whatever mechanism of L1 integration is effective in each case, taken together, these data indicate that the somatic integration of L1 elements induces the associated deletions.

### Megabase-size L1-mediated deletions cause loss of tumor-suppressor genes

**Fig. 5:**
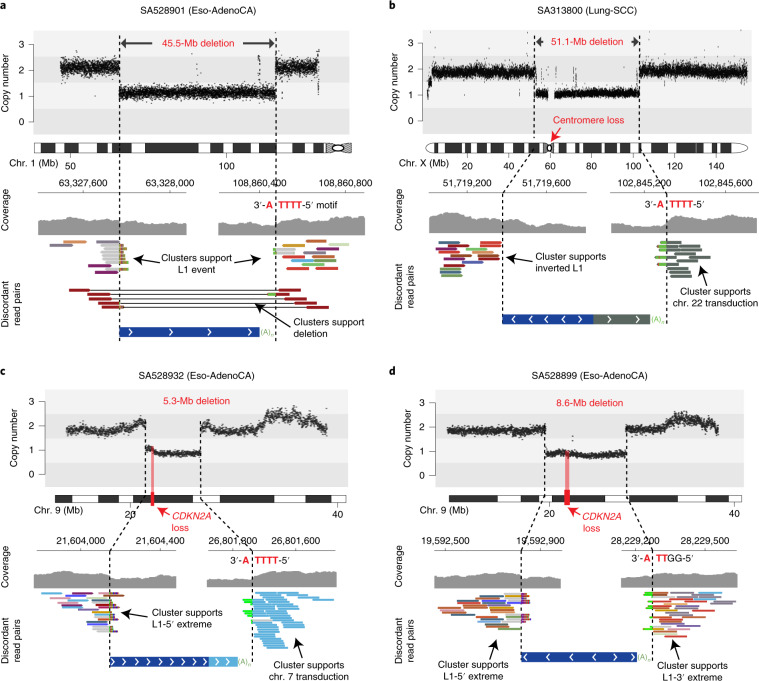
Somatic integration of L1 causes loss of megabase-size interstitial chromosomal regions in cancer. **a**, In esophageal adenocarcinoma sample SA528901, a 45.5-Mb interstitial deletion on chromosome 1 is generated after integration of a short L1 event. We observed a pair of clusters of discordant read pairs for which the mates support both extremes of the L1 insertion. Because the L1 element event is smaller than the library insert size, we also identified read pairs that span the L1 event and support the deletion. The L1-endonuclease 5′-TTTT/A-3′ motif identifies a target-primed reverse transcription (TPRT) L1-integration mechanism. **b**, In esophageal tumor sample SA313800, a partnered transduction^7^ (that is, the transduced region and its companion L1 source element) from chromosome 22 is integrated on chromosome X, promoting a 51.1-Mb deletion that removes the centromere. One negative cluster (green reads) supports a small region transduced from chromosome 22. **c**, L1-mediated deletions promote the loss of tumor-suppressor genes. In esophageal tumor sample SA528932, the somatic integration on chromosome 9 of a partnered transduction from chromosome 7, promotes a 5.3-Mb deletion that involves the loss of one copy of the tumor-suppressor gene *CDKN2A*. We observed a positive cluster of reads for which the mates map onto the 5′ extreme of an L1, and a negative cluster that contains split reads that match a poly(A) region and for which the mates map onto a region that is transduced from chromosome 7 (light blue). **d**, In a second esophageal adenocarcinoma sample, SA528899, the integration of an L1 retrotransposon generates an 8.6-Mb deletion that involves the same tumor-suppressor gene, *CDKN2A*. The sequencing data reveal two clusters—positive and negative—for which the mates support the L1 event.

Most L1-mediated deletions ranged from a few hundred to thousands of base pairs, although occasionally megabase-long regions of a chromosome were deleted (Fig. 4c and Supplementary Table 6). For example, in esophageal tumor sample SA528901, we found a 45.5-Mb interstitial deletion that involved the p31.3–p13.3 regions of chromosome 1 (Fig. 5a), in which both breakpoints of the rearrangement showed the hallmarks of a deletion mediated by integration of an L1 element. Here, the L1 element is 5′ truncated, which generated a small L1 insertion, allowing a fraction of the sequencing read pairs to span both breakpoints of the rearrangement. This unequivocally supports the model that the observed copy-number change is indeed a deletion mediated by retrotransposition of an L1 element. Similarly, in a lung tumor sample, SA313800, we found an interstitial L1-mediated deletion that induced the loss of 51.1 Mb from chromosome X, which included the centromere (Fig. 5b).

L1-mediated deletions were, on occasion, driver events and caused the loss of tumor-suppressor genes. In esophageal tumor sample SA528932, the integration of an L1 transduction from chromosome 7p12.3 to the short arm of chromosome 9 caused a 5.3-Mb clonal deletion that involved the 9p21.3–9p21.2 region. This led to the loss of one copy of a key tumor-suppressor gene, *CDKN2A* (Fig. 5c), which is deleted in many cancer types including esophageal tumors^33–36^. Notably, the sequencing data revealed a somatic transduction that arose from this L1 element at its new insertion site, demonstrating that L1 events that promote deletions can be competent for retrotransposition (Supplementary Fig. 13). In a second esophageal tumor sample, SA528899, an L1 element integrated into chromosome 9 promoted an 8.6-Mb clonal deletion that encompasses the 9p22.1–9p21.1 region that removes one copy of the same tumor-suppressor gene, *CDKN2A* (Fig. 5d). Thus, L1-mediated deletions have clear oncogenic potential.

### L1 retrotransposition generates other types of structural variation in human tumors

**Fig. 6:**
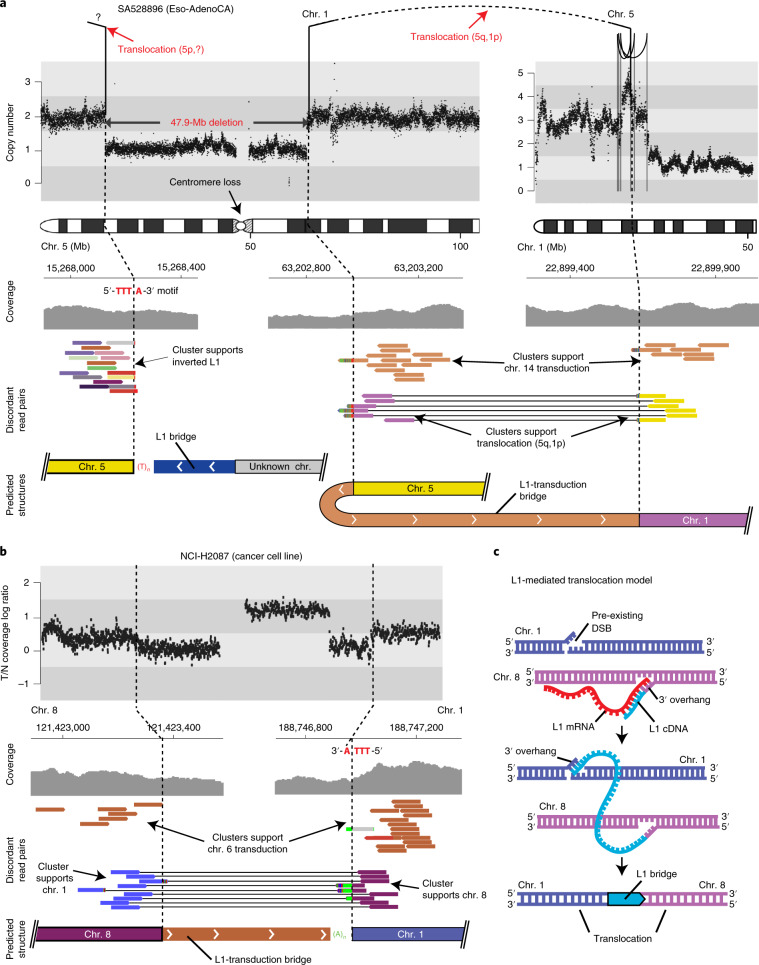
Somatic L1 integration promotes translocations in human cancers. **a**, In esophageal adenocarcinoma sample SA528896, two separate L1 events mediate interchromosomal rearrangements. In the first, an L1 transduction from a source element on chromosome 14q23.1 bridged an unbalanced translocation from chromosome 1p to 5q. A second somatic retrotransposition event bridged from chromosome 5p to an unknown part of the genome, completing a 47.9-Mb interstitial copy-number loss on chromosome 5 that removes the centromere. **b**, In a cancer cell line, NCI-H2087, we found an interchromosomal translocation, between chromosomes 8 and 1, mediated by a region transduced from chromosome 6, which acts as a bridge and joins both chromosomes. We observed two read clusters, positive and negative, that demarcate the boundaries of the rearrangement, for which the mates support the transduction event. In addition, two reciprocal clusters span the insertion breakpoints, supporting the translocation between chromosomes 8 and 1. **c**, A model for megabase-size L1-mediated interchromosomal rearrangements. L1-endonuclease cleavage promotes a 3′ overhang in the negative strand, retrotranscription starts and the cDNA (−) strand invades a second 3′ overhang from a pre-existing double-strand break on a different chromosome, leading to translocation.

Somatic retrotransposition can also be involved in mediating or repairing more complex structural variants. In one esophageal tumor sample, SA528896, two separate L1-mediated structural variants were present within a complex cluster of rearrangements (Fig. 6a). In the first, an L1 transduction from a source element on chromosome 14q23.1 bridged an unbalanced translocation from chromosome 1p to 5q. A second somatic retrotransposition event bridged from chromosome 5p to an unknown part of the genome, completing a large interstitial copy-number loss on chromosome 5 that involves the centromere. This case suggests that retrotransposon transcripts and their reverse-transcriptase machinery can mediate breakage and repair of complex dsDNA breaks, spanning two chromosomes.

To explore this further, we identified single-L1 clusters with no reciprocal cluster in the cancer cell lines that were sequenced by using mate pairs with 3 kb and 10 kb inserts. Such events may correspond to hidden genomic translocations leading to the linkage of two different chromosomes, in which L1 retrotransposition is involved. One of the samples, NCI-H2087, showed translocation breakpoints at 1q31.1 and 8q24.12, both of which had the hallmarks of L1-mediated deletions, for which the mate-pair sequencing data identified an orphan L1 transduction from chromosome 6p24 that bridged both chromosomes (Fig. 6b). The configuration has also been confirmed by using long-read single-molecule sequencing (Supplementary Fig. 11). This interchromosomal rearrangement is likely mediated by the aberrant operation of L1-integration mechanism, in which the L1-transduced cDNA is wrongly paired with a second 3′ overhang from a pre-existing double-strand break generated in a second chromosome^32^ (Fig. 6c).

**Fig. 7:**
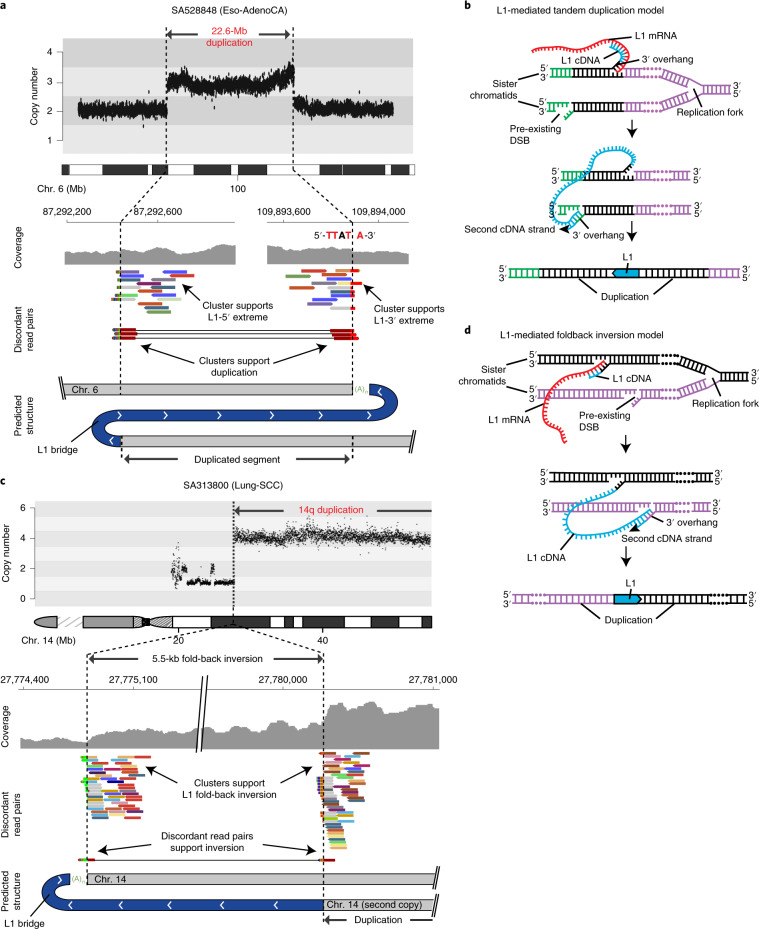
Somatic L1 integration promotes duplications of megabase-scale regions in human cancers. **a**, In esophageal adenocarcinoma sample SA528848, we found a 22.6-Mb tandem duplication on the long arm of chromosome 6. The analysis of the sequencing data at the boundaries of the rearrangement breakpoints reveals two clusters of discordant read pairs for which the mates support the involvement of an L1 event. Because the L1 element was shorter than the library size, we also found two reciprocal clusters that aligned 22.6 Mb apart on the genome and in opposite orientation, spanning the insertion breakpoints and confirming the tandem duplication. An L1-endonuclease 5′-TTT/A-3′ degenerate motif was found. **b**, Large direct tandem duplications can be generated if the cDNA (−) strand invades a second 3′ overhang from a pre-existing double-strand break that occurred on a sister chromatid, and downstream to the initial integration site locus. **c**, In lung tumor sample SA313800, a small L1 insertion causes a 79.6-Mb duplication of the 14q arm through the induction of a fold-back inversion rearrangement. The analysis of the sequencing data at the breakpoint revealed two clusters of discordant read pairs (multi-colored reads) with the same orientation, aligning close together (5.5 kb apart) and demarcating a copy-number change for which the sequencing density is much greater on the right half of the rearrangement than the left. Both clusters of multi-colored reads support the integration of an L1. **d**, L1-mediated fold-back inversion model.

We also found evidence that L1 integrations can cause duplications of large genomic regions in human cancer. In esophageal tumor sample SA528848 (Fig. 7a), we identified two independent read clusters that support the integration of a small L1 event, coupled with a coverage drop at both breakpoints. Copy-number analysis revealed that the two L1 clusters demarcate the boundaries of a 22.6-Mb duplication that involves the 6q14.3–q21 region, suggesting that the L1 insertion could be the cause of such rearrangement by bridging sister chromatids during or after DNA replication (Fig. 7b). The analysis of the rearrangement data at the breakpoints identified read pairs that traverse the length of the L1 insertion breakpoints, and the L1-endonuclease motif is the L1 3′ insertion breakpoint, both confirming a single L1 event as the cause of a tandem duplication (Fig. 7a). Notably, this duplication increases the copy number of the cyclin C gene, *CCNC*, which is dysregulated in some tumors^37^.

### L1-mediated rearrangements can induce breakage–fusion–bridge cycles that trigger oncogene amplification

L1 retrotranspositions can also induce genomic instability by triggering breakage–fusion–bridge cycles. This form of genetic instability starts with end-to-end fusion of broken sister chromatids, and lead to a dicentric chromosome that forms an anaphase bridge during mitosis. Classically, the end-to-end chromosome fusions are thought to arise from telomere attrition^38–40^. We found, however, that somatic retrotransposition can induce the first inverted rearrangement that generates end-to-end fusion of sister chromatids. In lung tumor sample SA313800 (Fig. 7c), we found a small L1 event inserted on chromosome 14q that demarcates a copy-number change that involves a 79.6-Mb amplification of the 14q arm. Analysis of the sequencing data at the breakpoint revealed two discordant read clusters with the same orientation, which are 5.5 kb apart and support the integration of an L1. Both discordant clusters demarcate an increment of the sequencing coverage, for which the density is much greater in the right cluster. The only genomic structure that can explain this pattern is a fold-back inversion in which the two sister chromatids are bridged by an L1 retrotransposition in head-to-head (inverted) orientation (Fig. 7d).

**Fig. 8:**
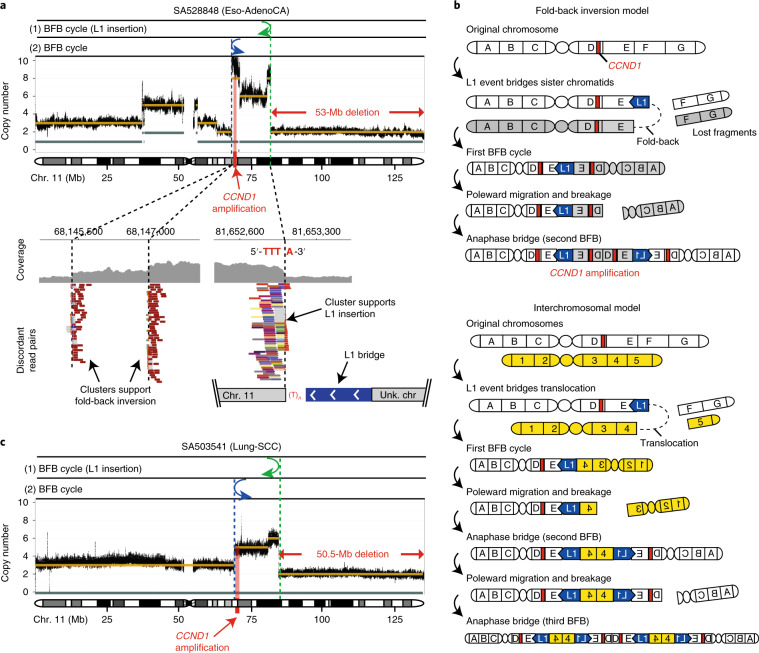
Somatic integration of L1 can trigger breakage–fusion–bridge cycles that lead to oncogene amplification. **a**, In esophageal adenocarcinoma sample SA528848, a single cluster of discordant reads (multi-colored reads) together with an L1-endonuclease cleavage site motif 5′-TTT/A-3′ supports the integration of an L1 event that demarcates a 53-Mb telomeric (that is, including the telomere) deletion, from a region of massive amplification that involves *CCND1*. Around 14 Mb upstream of the breakpoint of the deletion, we observed the presence of two clusters of read pairs (brown reads) that align close together and in the same orientation, which demarcate a change in copy number; this is a distinctive pattern of a fold-back inversion^42,43^, a rearrangement typically found to be associated with breakage–fusion–bridge (BFB) repair. In this fold-back inversion, the coverage shows much greater density on the right half of the rearrangement than the left, indicating that the abnormal chromosome is folded back on itself leading to duplicated genomic sequences in a head-to-head (inverted) orientation. The patterns described here suggest two independent breakage–fusion–bridge cycles, marked with (1) and (2). The copy-number plot shows the consensus total copy numbers (gold band) and the minor allele copy numbers (gray band). **b**, Models for the patterns described in **a**. The fold-back inversion model involves two breakage–fusion–bridge cycles, one induced by L1-mediated fold-back inversion (see Fig. 7d), and a second induced by standard breakage–fusion–bridge repair. The interchromosomal rearrangement model involves an interchromosomal rearrangement mediated by an L1, followed by one extra cycle of breakage–fusion–bridge repair. **c**, In lung cancer sample SA503541, the integration of an L1 retrotransposon is associated with a 50-Mb loss on 11q that includes the telomere, and activates breakage–fusion–bridge repair, which leads to the amplification of *CCND1*.

In the example described above (Fig. 7c,d), no further breaks occurred, and the L1 retrotransposition generated an isochromosome (14q). In addition, we found examples in which the fusion of two chromatids by an L1 bridge induced further cycles of breakage–fusion–bridge repair. In esophageal tumor sample SA528848, we identified a cluster of reads on the long arm of chromosome 11 that had the typical hallmarks of an L1-mediated rearrangement (Fig. 8a). Copy-number data analysis showed that this L1 insertion point demarcated a 53-Mb deletion, which involved the loss of the telomeric region, from a region of massive amplification on chromosome 11. The amplified region on chromosome 11 contains the *CCND1* oncogene, which is amplified in many human cancers^41^. The other end of this amplification was bound by a conventional fold-back inversion rearrangement (Fig. 8a), which is indicative of breakage–fusion–bridge repair^42,43^.

These patterns suggest the following sequence of events. During or soon after S phase, a somatic L1 retrotransposition bridges across sister chromatids in inverted orientation, breaking off the telomeric ends of 11q, which are then lost to the clone during the subsequent cell division (fold-back inversion model, Fig. 8b). The chromatids bridged by the L1 insertion now produce a dicentric chromosome. During mitosis, the two centromeres are pulled to opposite poles of the dividing cell, creating an anaphase bridge, which is resolved by further dsDNA breakage. This induces a second cycle of breakage–fusion–bridge repair, albeit not one mediated by L1 retrotransposition. These cycles lead to rapid-fire amplification of the *CCND1* oncogene. Alternatively, an interchromosomal rearrangement mediated by L1 retrotransposition (interchromosomal rearrangement model, Fig. 8b) followed by two cycles of breakage–fusion–bridge repair could generate similar copy-number patterns with telomere loss and amplification of *CCND1*.

Our data show that L1-mediated retrotransposition is an alternative mechanism of creating the first dicentric chromosome that induces subsequent rounds of chromosomal breakage and repair. If this occurs near an oncogene, the resulting amplification can provide a powerful selective advantage to the clone. We searched the PCAWG dataset for other rearrangements that included copy-number amplifications from telomeric deletions that were mediated by L1 integration. We found four more such events across three cancer samples (Supplementary Fig. 14). In a lung tumor sample, SA503541, we found almost identical rearrangements to the one described above (Fig. 8c). In this case, a somatic L1 event also generated telomere loss that induced a second cycle of breakage–fusion–bridge repair. The megabase-size amplification of chromosomal regions also targeted the *CCND1* oncogene, in which the boundaries were demarcated by the L1 insertion breakpoint and a fold-back inversion, which indicates breakage–fusion–bridge repair. The independent occurrence of these patterns, which involve the amplification of *CCND1*, in two different tumor samples (SA528848 and SA503541) demonstrates a mutational mechanism mediated by L1 retrotransposition, which likely contributes to the development of human cancer.

## Discussion

Here we characterize the patterns and mechanisms of cancer retrotransposition on a multidimensional scale, across 2,954 cancer genomes, integrated with rearrangement, transcriptomic and copy-number data. Our analyses provide a new perspective on the long-standing question of whether the activation of retrotransposons is relevant in human oncogenesis. Our findings demonstrate that major restructuring of cancer genomes can sometimes emerge from aberrant L1 retrotransposition events in tumors with high retrotransposition rates, particularly in esophageal, lung and head-and-neck cancers. L1-mediated deletions can promote the loss of megabase-scale regions of a chromosome that may involve centromeres and telomeres. It is likely that the majority of such genomic rearrangements would be harmful for a cancer clone. However, occasionally, L1-mediated deletions may promote cancer-driving rearrangements that involve the loss of tumor-suppressor genes and/or the amplification of oncogenes, representing another mechanism by which cancer clones acquire new mutations that help them to survive and grow. We expect that structural variants induced by somatic retrotransposition in human cancer are more frequent than we could unambiguously characterize here, given the constraints on the fragment sizes of paired-end sequencing libraries. Long-read sequencing technologies should be able to provide a more comprehensive picture of how frequent such events are. Relatively few germline L1 loci in a given tumor, typically one to three copies, are responsible for such marked structural remodeling. Given the role that these L1 copies may have in some cancer types, this work underscores the importance of characterizing cancer genomes for patterns of L1 retrotransposition.

## Methods

### Pan-cancer datasets

#### Whole-genome sequencing dataset

We analyzed Illumina whole-genome paired-end sequencing reads (100–150 bp) from 2,954 tumors and matched normal samples across 38 cancer types^21^. On the basis of the robustness of the retrotransposition calls (false discovery rate of <5%), we opted to retain all samples that were preliminarily excluded by the PCAWG Consortium^21^, as they were excluded from SNV and structural variation analyses on the basis of read direction biases from PCR artifacts or poor sequence quality, but were not found to be problematic for retrotransposition analysis. For the majority of donors, the tumor specimens consisted of a fresh frozen sample, whereas the normal specimens consisted of a blood sample. Most of the tumor samples came from treatment-free primary cancers, although there was also a small number of donors with multiple samples of primary, metastatic and/or recurrent tumors. The average coverage was 30 reads per genome for normal samples, whereas tumor samples had a bimodal coverage distribution with maxima at 38 and 60 reads per bp (Supplementary Fig. 1 and Supplementary Table 1). BWA-mem^44^ v.0.7.8-r455 was used to align each tumor and normal sample to human reference build GRCh37. Additional technical details of the sequencing metrics are provided in Supplementary Table 1 and in the PCAWG lead paper^21^. The Ethics oversight for the PCAWG protocol was undertaken by the TCGA Program Office and the Ethics and Governance Committee of the ICGC.

#### Transcriptome dataset

About half of the donors (1,188) with whole-genome data in PCAWG had at least one tumor specimen with whole transcriptome obtained by RNA sequencing (RNA-seq). Mapping onto the reference was carried out using two independent read aligners, STAR^45^ v.2.4.0i, two-pass and TopHat2 (ref. ^46^) v.2.0.12. Gene expression was quantified with HTSeq^47^ v.0.6.1p1 and consensus normalized expression values, in fragments per kilobase of transcript per million mapped reads (FPKM), were obtained by averaging the expression from STAR and TopHat2. A more detailed description of RNA-seq data processing is provided by the PCAWG Integration of Transcriptome and Genome Working Group^48^.

#### Copy-number dataset

We analyzed copy-number profiles obtained by the PCAWG Evolution and Heterogeneity Working Group, using a consensus approach that combines six different state-of-the-art copy-number calling methods^49^. GC content corrected logR values were extracted using the Battenberg algorithm^50^, smoothed using a running median and transformed into copy-number space according to *n* = [(2(1 − *ρ*) + *ψρ*)2^logR^ − 2(1 − *ρ*)]/*ρ* where *ρ* and *ψ* are consensus tumor purity and ploidy, respectively.

#### Structural variant dataset

The structural variation call set was generated by the PCAWG Somatic Structural Variation Working Group by merging the structural variant calls from four independent calling pipelines^51^. The merged structural variant calls were further required to have a consistent change in copy number.

### Analysis of somatic retrotransposition

#### Detection of mobile element insertions using TraFiC-mem

BAM files from tumor and matched normal pairs were processed with TraFiC-mem v.1.1.0 to identify somatic mobile element insertions (MEIs) including solo-L1, L1-mediated transductions, Alu, SVA and ERV-K using Illumina paired-end mapping data. TraFiC-mem starts by identifying candidate somatic MEIs by analyzing discordant read pairs. In contrast to a previous version of the algorithm^7^, the new pipeline uses BWA-mem v.0.7.17 instead of RepeatMasker as the search engine for the identification of retrotransposon-like sequences in the sequencing reads. Calls obtained at this step are preliminary, in which MEI features are outlined and insertion coordinates represent ranges that surround the breakpoints. Then, a new module of TraFiC-mem, called MEIBA (from Mobile Element Insertion Breakpoint Analyzer), is used to identify the integration breakpoints to base-pair resolution and to perform a detailed characterization of MEI features, including structure, subfamily assignment and insertion site annotation. TraFiC-mem is illustrated in Supplementary Fig. 3. Detailed information about the pipeline is provided in the Supplementary Note.

#### Identification of germline and somatic L1 source elements

Because L1-mediated transductions are defined by the retrotransposition of unique, non-repetitive genomic sequences, we can unambiguously identify the L1 source element from which they derive^7^. The method relies on the detection of unique DNA regions retrotransposed somatically elsewhere in the cancer genome from a single locus that matches the 10-kb downstream region of a reference full-length L1 element or a putative non-reference polymorphic L1 element detected by TraFiC-mem across the matched normal samples in the PCAWG cohort^21^. When transduced regions were derived from the downstream region of a putative L1 event present in the tumor genome but not in the matched normal genome, we catalogued these elements as somatic L1 source loci.

#### Identification of processed pseudogene insertions

An additional separate module of TraFiC-mem was implemented for the identification of somatic insertions of processed pseudogenes. The method relies on the same principle as for the identification of somatic MEI events, through the detection of two reciprocal clusters of discordant read pairs, namely positive and negative, that supports an insertion event in the reference genome. However, the method differs from standard MEI calling to which the read mates map, as in this case mates are required to map to exons that belong to the same source protein-coding gene in GENCODE v.19. To avoid misclassification with standard genomic rearrangements that involve coding regions, we use MEIBA—described above—to reconstruct the insertion breakpoint junctions looking for hallmarks of retrotransposition, including the poly(A) tract and duplication of the target site. Candidate insertions without a poly(A) tail were discarded.

#### Identification of L1-mediated deletions

Independent read clusters, identified with TraFic-mem, supporting an L1 event (that is, clusters of discordant read pairs with no apparent reciprocal cluster within the proximal 500 bp, and for which the mates support a somatic L1 retrotransposition event) were interrogated for the presence of an associated change in copy number in its proximity. In brief, we looked for copy-number loss calls from the PCAWG Evolution and Heterogeneity Working Group for which the following conditions were fulfilled: (1) the upstream breakpoint matches an independent L1 cluster in positive orientation, (2) the corresponding downstream breakpoint, if any, from the same change in copy number matches an independent L1 cluster in negative orientation, and (3) the reconstruction of the structure of the putative insertion causing the deletion is compatible with one-single retrotransposition event. We used MEIBA (Supplementary Note) to reconstruct the insertion breakpoint junctions to confirm the ends of the events and identify hallmarks of retrotransposition, including the poly(A) tract and duplication of the target site.

An additional strategy was used for L1-mediated deletions that were shorter than 100 kb. Read-depth drops in the proximity of independent clusters were detected by, first, normalizing the read depth on each side of the cluster, using the matched normal sample as a reference. Then, the ratio between the normalized read depth on both sides of the cluster was computed for windows of 200–5,000 bp. Adjacent buffer regions of 300 bp on each side of the cluster were omitted from read-depth calculations to avoid false positives caused by sequence repeats. Pairs of independent reciprocal (positive–negative) clusters were selected such that: (1) both clusters were located less than 100 kb apart, (2) a potential drop in the read-depth ratio was identified, extending from the positive cluster to the negative cluster, and (3) the reconstruction of the structure of the putative insertion that caused the deletion was compatible with a single L1 event. For each cluster pair, the continuity and reliability of the copy-number drop was assessed by measuring the normalized read-depth ratio between non-overlapping 500-bp windows that spanned the region between the positive and negative clusters (that is the putative deletion) and windows upstream and downstream of the positive and negative clusters, respectively. The significance of each read-depth ratio drop was estimated nonparametrically using a null distribution of normalized read-depth ratios. This distribution was obtained for each tumor sample by randomly sampling 100,000 genomic locations (from copy-number segments showing the predominant copy number), and calculating read-depth ratios between both sides of each position. Nonparametric *P* values were calculated by comparing observed read-depth ratios with this null distribution, and adjusted using the Benjamini–Hochberg correction. Two cluster groups were produced: tier 1, pairs of reciprocal clusters with both clusters that had *P* < 0.1, and tier 2, pairs of reciprocal clusters with only one of both clusters having *P* < 0.1.

#### Retrotransposition rate enrichment and depletion across tumor types

For each tumor type with a minimum sample size of 15, we assessed whether it was enriched or depleted in retrotransposition compared to the overall retrotransposition burden using zero-inflated negative binomial regression, as implemented in the zeroinfl function of the pscl R package. This type of model takes into account the excess of zeros and the overdispersion that is present in this dataset. The MEI counts per sample were regressed on a binary factor that expressed whether they belonged to that particular type of cancer or to any other cancer type. On each regression, the magnitude and sign of the *z*-score indicates the effect size and directionality of the association. More specifically, positive *z*-scores indicate that a higher number of counts in the samples belongs to a particular cancer type compared with the rest (enrichment), whereas negative scores indicate a lower number of counts (depletion). Each *z*-score is accompanied by its *P* value to indicate the level of statistical significance.

#### Association between mutation in tumor-suppressor genes and retrotransposition and structural variantion rates

To assess whether the disruption of a particular tumor-suppressor gene was associated with a high level of retrotransposition, we used the whole-genome panorama of cancer driver events per sample produced by the PCAWG Drivers and Functional Interpretation Working Group^21^. This panorama includes coding and non-coding SNVs, insertions and deletions, copy-number alterations, structural variants and potentially predisposing germline variants. For each tumor-suppressor gene in the Cancer Gene Census database with mutational data, we stratified the samples into two groups—mutated tumor-suppressor genes and non-mutated tumor-suppressor genes. Then, we compared the distribution of MEI counts between both groups using a Mann–Whitney *U*-test to identify significant differences. *P* values were corrected for multiple testing using the Benjamini–Hochberg procedure. Adjusted *P* < 0.05 were considered significant. This analysis was done at both the level of the individual cancer type and the level of pan-cancer to identify tumor-type-specific associations. We further investigated whether there was a *TP53* dosage effect as follows: every PCAWG sample was classified into three groups according to *TP53* mutational status, namely wild-type, monoallelic and biallelic driver mutation. Then, the MEI counts distribution was compared for all possible group pair combinations using a Mann–Whitney *U*-test. The same analysis described above was applied to investigate the association between *TP53* mutation and other types of structural variation.

#### Correlation between L1 insertion and structural variation rate

For each sample, we computed the number of MEIs, the total number of structural variants and the number of five different structural variant classes: deletions, duplications, translocations, head-to-head inversions and tail-to-tail inversions, when data were available. Then, the correlation between the number of MEIs and the structural variant burden was assessed at both the level of the individual tumor type and the level of the pan-cancer using a Spearman’s rank test.

#### Association between L1 insertion rate and genomic features

The L1 insertion rate was calculated as the total number of somatic L1 insertions, identified across the complete PCAWG cohort per 1-Mb window. The density of L1 endonuclease motifs was computed as the number of canonical endonuclease motifs, here defined as TTTT|R (where R is A or G) or Y|AAAA (where Y is C or T) per 1-Mb window. To study the association of L1 insertion rate with multiple variables at single-nucleotide resolution, we used a statistical framework based on negative binomial regression, as described in detail previously^24^, and adapted to adjust for the genome-wide distribution of the L1 endonuclease motif; we stratified the genome into four bins (0–3) by the closeness of match to the motif. Bin 0 contains dissimilar DNA motifs, with four or five (out of five) mismatches, encompassing 1,150 Mb. Bins 1, 2 and 3 contain loci with three, two and at most one mismatches, encompassing 749 Mb, 380 Mb and 114 Mb, respectively. The closer match of either of the two DNA strands at each locus was considered. Histone mark data and DNase hypersensitivity data were obtained from the Roadmap Epigenomics Consortium by averaging the fold-enrichment signal across eight cell types and processed by stratifying into four genomic bins as described previously^24^: bin 0 contains regions with below-baseline signal (fold enrichment versus input <1), while bins 1–3 are approximately equal-sized bins that cover the remainder of the genome. RNA-seq data from Roadmap were processed by averaging across eight cell types; bin 0 contains non-expressed genes (FPKM = 0) and intergenic DNA not listed as expressed, while bins 1–3 included genes with up to 0.59, 5.68 and above 5.68 FPKM, respectively. Replication time was averaged across the eight ENCODE cell types and divided into four equal-sized genomic bins, where bin 0 is latest and bin 3 is earliest replicating. Essential genes were estimated from CRISPR screens in cell lines^52^. All enrichment scores shown compare bins 1–3 for a particular feature (replication time, histone marks, gene expression, L1 motif) versus bin 0 of the same feature. Bin 0 therefore always has log enrichment = 0 by definition and is not shown on plots. The analyses were restricted to regions of the genome with perfect CRG75 alignability.

#### Impact of retrotransposition insertions on gene expression

To study the transcriptional impact of a somatic L1 insertion within COSMIC cancer genes and promoters, we used RNA-seq data to compare gene-expression levels in samples with and without somatic L1 insertion. For each somatic L1 insertion within a cancer gene or promoter, we compared the gene FPKM between the sample having the insertion (study sample) and the remaining samples of the same tumor type (control samples). Using the distribution of gene-expression levels in control samples, we calculated the normalized gene expression differences using a Student’s *t*-test. To overcome the problems due to multiple testing, false discovery rate–adjusted *P* values (*q* values) were calculated using the Benjamini–Hochberg procedure, and adjusted *P* < 0.1 was considered to be significant.

#### Analysis of processed pseudogene expression

We analyzed the PCAWG RNA-seq data to identify and characterize the transcriptional consequences of somatic integrations of processed pseudogenes (PSD). We interrogated RNA-seq split reads and discordant read pairs, looking for chimeric retrocopies that involved PSDs and target genomic regions. For each PSD insertion somatic call, we extracted all of the RNA-seq reads (when available), mapping the source gene and the insertion target region, together with the RNA-seq unmapped reads for the corresponding sample. Then, we used these reads as a query in BLASTn^53^ v.2.7.1 searches against a database that contained all isoforms of the source gene described in RefSeq^54^, together with the genomic sequence ranging from −5 kb to +5 kb around the PSD integration site. Finally, we looked for RNA-seq discordant read pairs and/or RNA-seq clipped reads that supported the joint expression of PSD and target site. Only read pairs with one of the mates aligned to the host gene mRNA with >98% identity were considered. All expression signals were confirmed by visual inspection with Integrative Genomics Viewer v.2.4.10.

### Validation of somatic retrotransposition algorithms

#### In silico validation of TraFiC-mem

To evaluate the precision and recall of our algorithm TraFiC-mem, we reanalyzed a mock cancer genome into which we had previously seeded known somatic retrotransposition events at different levels of tumor clonality^7^. To create the artificial, tumoral genome, 10,000 L1 insertion breakpoints—including solo-L1, partnered and orphan transductions—were randomly distributed in the standard reference genome using BedTools v.2.25.0, of which 9,227 were inserted out of un-sequenced gaps. Then, ART^55^ (v.MountRainier-2016-06-05) was used to generate paired-end read sequencing data for both the standard and the artificial reference genomes to a 38× coverage. The simulation FASTQ files were aligned into the standard reference genome with BWA-mem^56 v.0.7.17^. Reads from the normal and tumor BAM files were randomly subsampled and merged with samtools v.1.7 at three distinct proportions to also produce tumor samples with 25%, 50% and 75% clonalities. After that, the four possible tumor and matched normal pairs were processed with TraFiC-mem to call MEIs. For each clonality, the identified MEIs were compared with the list of simulated MEIs to compute the number of true-positive (TP), false-positive (FP), true-negative (TN) and false-negative (FN) calls. Finally, precision and recall were computed as follows: Precision = TP/(TP + FP); Recall = TP/(TP + FN).

#### Validation of TraFiC-mem calls using single-molecule sequencing

We performed validation of 308 putative somatic retrotranspositions identified with TraFiC-mem in one cancer cell line (NCI-H2087) with high retrotransposition rate, and absent in the matched normal cell line (NCI-BL2087) derived from blood, by single-molecule sequencing using Oxford Nanopore technology. Genomic DNA was sheared to 10-kb fragments using Covaris g-TUBEs, and cleaned with 0.4× Ampure XP Beads. After end-repairing and dA-tailing using the NEBNext End Repair/dA-tailing module (NEB), whole-genome libraries were constructed with the Oxford Nanopore Sequencing 1D ligation library prep kit (SQK-LSK108, Oxford Nanopore Technologies). Genomic libraries were loaded on MinION R9.4 flowcells. We used the Oxford Nanopore basecaller Albacore v.2.0.1 to generate fastq files. After quality filtering of the fastq files and read trimming of the data with Porechop v.0.2.3, we used minimap2 (ref. ^57^) v.2.10-r764-dirty to map sequencing reads onto the hs37d5 reference genome. Sequencing coverages were 8.2× (NCI-BL2087) and 9.17× (NCI-H2087), and average read sizes of mapped reads were ~4.5 kb (NCI-BL2087) and ~11 kb (NCI-H2087). After obtaining the whole-genome BAM files for each of the 308 putative somatic retrotransposition calls identified with TraFic-mem, we interrogated the long-read tumor BAM file to find reads that validated the event. MEIs supported by at least one Nanopore read in the tumor and absent in the matched normal sample were considered true-positive somatic events, while MEIs not supported by long reads in the tumor and/or present in the matched normal were considered false-positive calls. Overall, we found 4.22% (13/308) false-positive events. False discovery rate (FDR) was estimated as follows: FDR = FP/(TP + FP).

#### Validation of L1-mediated rearrangements with PCR and single-molecule sequencing

We performed validation of 20 somatic L1-mediated rearrangements, mostly deletions, identified in two cancer cell lines with high retrotransposition rates (NCI-H2009 and NCI-H2087). We carried out PCR followed by single-molecule sequencing of amplicons from the two tumor cell lines and their matched normal samples (NCI-BL2009 and NCI-BL2087) using a MinION from Oxford Nanopore. PCR primers were designed to amplify three regions from each event (namely, 5′-extreme, 3′-extreme and target sites) as shown in Supplementary Fig. 10.

#### Validation of L1-mediated rearrangements using mate pairs

To further validate and characterize L1-mediated rearrangements, we performed 10× mate-pair whole-genome sequencing using libraries with two different insert sizes (4 kb and 10 kb), which can span the integrated L1 element that caused the deletion, enabling the validation of the involvement of L1 in the generation of such rearrangements. Mate-pair reads (100 nucleotides long) were aligned to the human reference with BWA-mem v.0.7.17. Then, for each candidate L1-mediated rearrangement, we searched for discordant mate-pair clusters that spanned the breakpoints and supported the L1-mediated event. Each event was confirmed by visual inspection of the BAM files using Integrative Genomics Viewer v.2.4.10.

### Reporting Summary

Further information on research design is available in the Nature Research Reporting Summary linked to this article.

## Online content

Any methods, additional references, Nature Research reporting summaries, source data, extended data, supplementary information, acknowledgements, peer review information; details of author contributions and competing interests; and statements of data and code availability are available at 10.1038/s41588-019-0562-0.

## Supplementary information

Supplementary Information Supplementary Figs. 1–14 and Notes

Reporting Summary

Supplementary Tables Supplementary Tables 1–8

## Data Availability

Somatic and germline variant calls, mutational signatures, subclonal reconstructions, transcript abundance, splice calls and other core data generated by the ICGC/TCGA PCAWG Consortium are available for download at https://dcc.icgc.org/releases/PCAWG. Additional information on accessing the data, including raw read files, can be found at https://docs.icgc.org/pcawg/data/. In accordance with the data access policies of the ICGC and TCGA projects, most molecular, clinical and specimen data are in an open tier, which does not require access approval. To access potentially identifying information, such as germline alleles and underlying sequencing data, researchers will need to apply to the TCGA Data Access Committee (DAC) via dbGaP (https://dbgap.ncbi.nlm.nih.gov/aa/wga.cgi?page=login) for access to the TCGA portion of the dataset, and to the ICGC Data Access Compliance Office (DACO; http://icgc.org/daco) for the ICGC portion. In addition, to access somatic SNVs derived from TCGA donors, researchers will also need to obtain dbGaP authorization. In addition, the analyses in this paper used a number of datasets that were derived from the raw sequencing data and variant calls (Supplementary Table 8). The individual datasets are available at Synapse (https://www.synapse.org/), which are also mirrored at DCC portal (https://dcc.icgc.org). Full links, filenames, accession numbers and descriptions are detailed in Supplementary Table 8. VCF files for somatic mobile element insertions described specifically in this manuscript can be found at Synapse, under accession number syn21052009, and in DCC portal at https://dcc.icgc.org/releases/PCAWG/retrotransposition.

## Code availability

The core computational pipelines used by the PCAWG Consortium for alignment, quality control and variant calling are available to the public at https://dockstore.org/search?search=pcawg under a GNU General Public License v.3.0, which allows for reuse and distribution. The algorithm for the identification of somatic retrotransposition events (TraFiC-mem) is available at https://gitlab.com/mobilegenomesgroup/TraFiC (v.1.2.0).
